# Persistent Directed Flag Laplacian (PDFL)-Based Machine Learning for Protein–Ligand Binding Affinity Prediction

**DOI:** 10.1021/acs.jctc.5c00074

**Published:** 2025-04-05

**Authors:** Mushal Zia, Benjamin Jones, Hongsong Feng, Guo-Wei Wei

**Affiliations:** † Department of Mathematics, Michigan State University, East Lansing, Michigan 48824, United States; ‡ Department of Electrical and Computer Engineering, Michigan State University, East Lansing, Michigan 48824, United States; § Department of Biochemistry and Molecular Biology, Michigan State University, East Lansing, Michigan 48824, United States

## Abstract

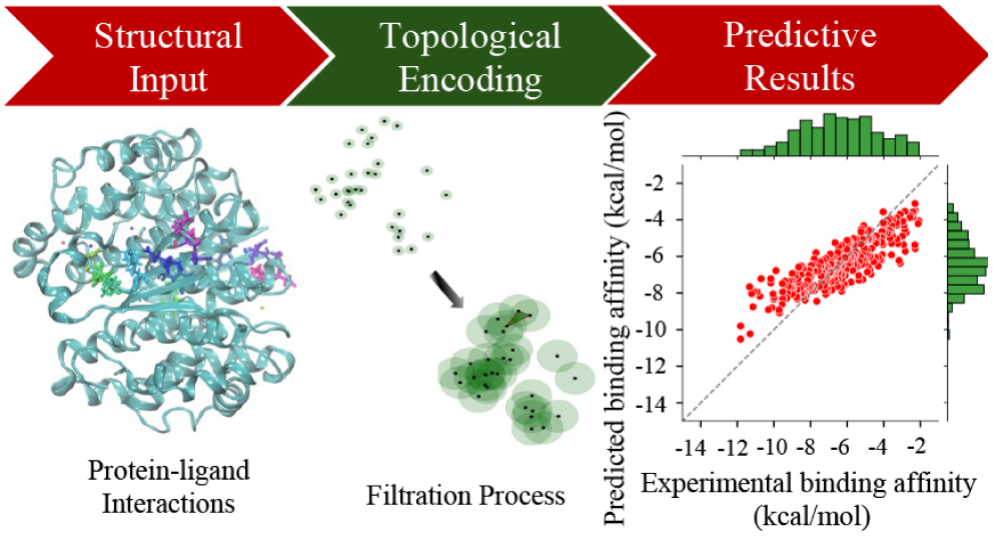

Directionality in molecular and biomolecular networks plays an important role in the accurate representation of the complex, dynamic, and asymmetrical nature of interactions present in protein–ligand binding, signal transduction, and biological pathways. Most traditional techniques of topological data analysis (TDA), such as persistent homology (PH) and persistent Laplacian (PL), overlook this aspect in their standard form. To address this, we present the persistent directed flag Laplacian (PDFL), which incorporates directed flag complexes to account for edges with directionality originated from polarization, gene regulation, heterogeneous interactions, etc. This study marks the first application of PDFL, providing an in-depth analysis of spectral graph theory combined with machine learning. In addition to its superior accuracy and reliability, the PDFL model offers simplicity by requiring only raw inputs without complex data processing. We validated our multikernel PDFL model for its scoring power against other state-of-the-art methods on three popular benchmarks, namely PDBbind v2007, v2013, and v2016. The computational results indicate that the proposed PDFL model outperforms competitors in protein–ligand binding affinity predictions, suggesting that PDFL is a promising tool for protein engineering, drug discovery, and general applications in science and engineering.

## 1 Introduction

Protein–ligand binding is a crucial interaction in which a protein, such as an enzyme or receptor, binds to one or more ligands. This interaction is fundamental to various biological processes, including cell signaling, molecular transport, and metabolism. Although proteins typically bind to only certain ligands because of their specific structural, electrostatic, and chemical compatibility, such as in hormone-receptor binding and enzyme–substrate interactions, there are cases where a protein binds to multiple small molecules. Protein–ligand binding is typically governed by noncovalent forces, including hydrogen bonds, van der Waals forces, hydrophobic interactions, electrostatic interactions, and ionic bonds. Under physiological conditions, hydrophobic residues on the protein interact with the nonpolar ligand to stabilize the binding. In drug discovery, many pharmaceuticals are designed to bind to specific proteins, such as receptors or enzymes, to modulate their functions. Moreover, protein–ligand interactions are also exploited in biosensors, diagnostic strategies, and protein engineering.

Experimental studies on protein–ligand binding are expensive. Computer modeling of protein–ligand binding is essential for understanding molecular interactions and plays a significant role in drug discovery. Various computational methods have been developed to predict the binding modes, affinities, and thermodynamic properties of these interactions. Among them, machine learning predictions have become very popular in the past decade^1−3^ due to their ability to incorporate fast-growing experimental data for accurate predictions. Mathematical artificial intelligence (Math AI) paradigms^4,5^ such as topological deep learning (TDL)^6^ have been introduced for protein–ligand binding prediction.^7,8^ This approach secured some of the best results in D3R Grand Challenges, an annual worldwide competition series in computer-aided drug design.^4,5^

One of the key components of TDL is persistent homology (PH)^9,10^ an algebraic topology tool for topological data analysis (TDA). It is well recognized that the foundational idea behind TDA is that data exhibit a discernible shape regardless of its complexity, and that shape matters. In other words, the topological or geometric structure of the data can reveal significant key patterns and relationships that may not be instantly recognizable from raw data alone. TDA utilizes PH to study and track features on varying scales by merging concepts from algebraic topology and data science, thus revealing insights into the connectivity and geometry of the underlying data that cannot be obtained from traditional mathematical, statistical and physical methods.^11^ The strength of TDA lies in its ability to encompass essential features through topological signatures while filtering out irrelevant ones, thus allowing the simplification of data into a more fitting form for analysis. These signatures are succinct mathematical representations of topological features such as connectivity, loops, voids, and higher-dimensional analogues, which capture the shape and underlying organization of complex data. Betti numbers count the number of these topological features in various dimensions; for instance, a loop captured by a one-dimensional Betti number indicates the presence of a closed pathway or feedback cycle in space. Similarly, a persistent Betti number provides information about the evolution of these topological features on varying scales.

While PH and other algebraic topological techniques have successfully helped simplify complex molecular structures and reduce dimensions in biological systems^12^ these tools struggle with tracking nontopological shape evolution. The persistent combinatorial Laplacian, or persistent Laplacian (PL), was introduced in 2019 to address some of the limitations of PH.^13^ PL can be regarded as a generalization of the classical graph Laplacian to higher topological dimensional complex structures and as an extension of PH to the nonharmonic spectra, as its harmonic spectra recover the topological invariants. Moreover, PL may be considered as the counterpart of persistent Hodge Laplacian, which is defined on differentiable manifolds.^14^ The persistent Hodge Laplacian uses differential forms, rather than simplicial complexes, to study and analyze manifolds, cohomology, and topological invariants in higher-dimensional spaces. PL has stimulated great theoretical interest^15−18^ and has had significant applications^19^ including protein engineering^20^ and accurate prediction of emerging dominant virus variants.^21^ A review of this trending topic is available in literature.^22^

In further advancement, following the framework of persistent directed flag complex homology^23^ Jones and Wei recently proposed a new TDA tool called the persistent directed flag Laplacian (PDFL).^24^ The directed flag Laplacian neglects multiscale analysis and thus has limited utility for data analysis. PDFL extends the concept of persistent Laplacian to a persistent directed flag complex, offering a new topological tool for modeling gene regulation, directed graph, atomic polarization, etc. This extension allows for a more comprehensive analysis, particularly in directed systems, by introducing a spatial dimension through the filtration parameter. The directed flag Laplacian provides a deeper analysis of multiscale topological features in directed networks by acting on simplicial complexes and directed graphs (digraphs). Moreover, it expands the ability of TDA by incorporating comprehensive insights into complex molecular structures, thus enabling the handling of directional network data, which can be challenging at times.

In this work, we present the first application of PDFL by integrating spectral graph theory with flexibility-rigidity index (FRI) based methods^25^ allowing advanced machine learning techniques such as gradient boost decision trees (GBDT) with topological atomic descriptors. This framework accounts for the directionality of protein–ligand interactions on the basis of their electronegativity differences and is used to develop multikernel predictive models tested against three large data sets. In the following sections, we explore the mathematical foundations, computational implementation, and impressive performance of our PDFL model across multiple benchmark data sets.

## 2 Results and Discussion

### 2.1 An Overview to the Persistent Directed Flag Laplacian Learning

**Figure 1:**
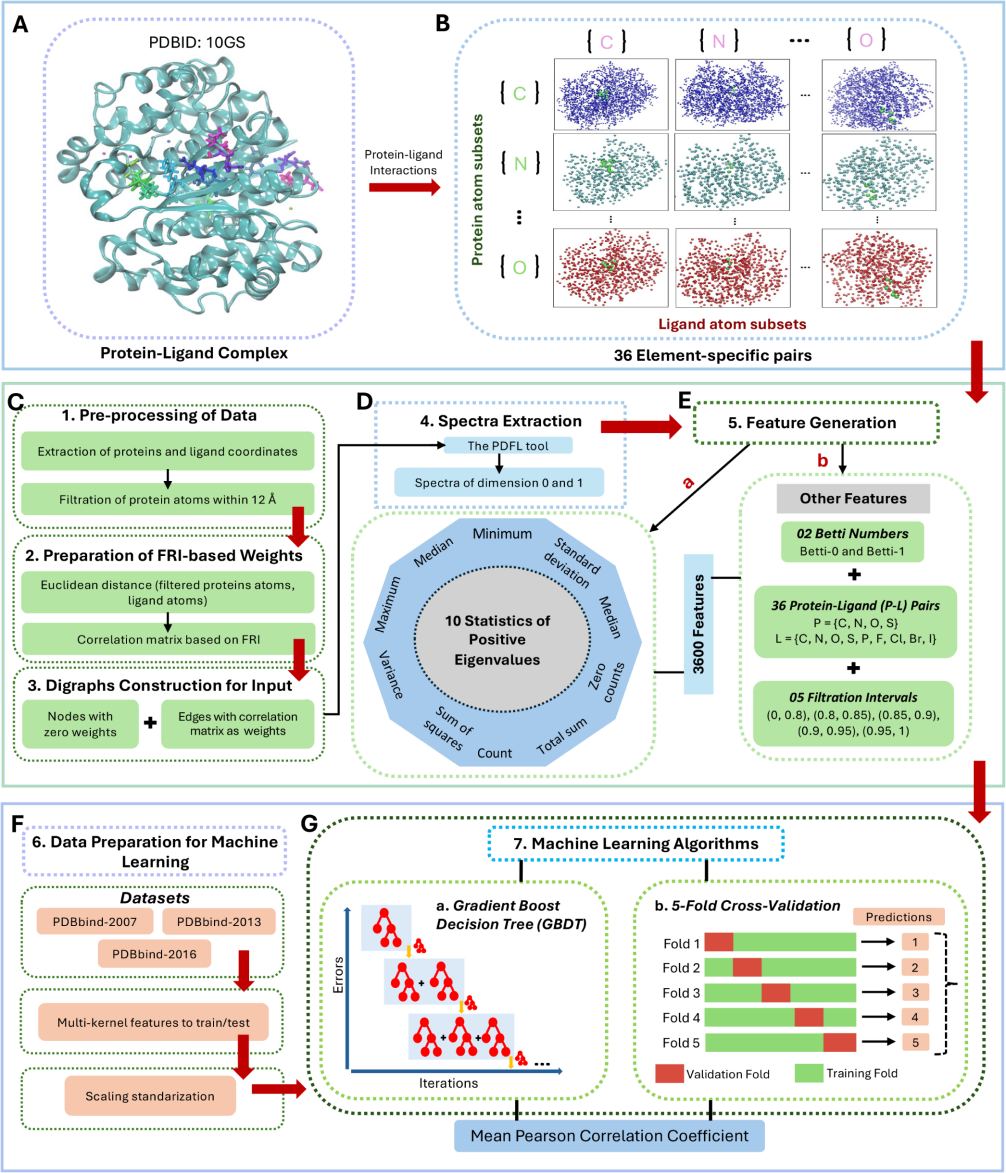
Illustration of workflow for protein–ligand binding affinity predicitions through Persistent Directed Flag Laplacian (PDFL) learning. (A) A protein–ligand complex with PDBID 10GS is shown. (B) A graphical portray of 36 element-specific pairs formed by four protein atoms and 9 ligand atoms representing their interactions. (C-E) An outline of data processing and input preparation for PDFL to generate spectra is shown. A total of 3600 features are generated for each protein–ligand complex by a combination of 36*[Element-types]*5[Intervals]*2[Betti numbers]*10[Statistical features]. (F, G) Three data sets from the Protein Data Bank (PDB) are used in this work to train the PDFL model with machine learning techniques, such as Gradient Boost Decision Tree (GBDT) and 5-fold Cross-Validation. Mean Pearson correlation coefficient is calculated for a number of predictions which shows the strength of protein–ligand binding affinity.

The persistent directed flag Laplacian (PDFL) is built by extending the software Flagser^23^ and leveraging its core functionalities to generate directed flag complexes and the corresponding (co)boundary matrices. PDFL computes the spectra of Laplacian matrices associated with directed flag complexes by taking weighted and filtered digraphs as an input (Figure 1C). In the present work, an element-level multiscale weighted rigidity index  is considered that captures the total interaction strength between all pairs of protein atoms *p* ∈ *P* and ligand atoms *l* ∈ *L* within a predefined cutoff distance *c* and is defined as
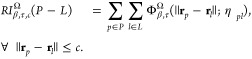
1

Here, ||**r**_*p*_ - **r**_*l*_|| represents the Euclidean distance, and η_*pl*_ is the characteristic distance between atoms *p* and *l*. Moreover, Ω = *E* or Ω = *L* denotes the kernel index with β as the kernel order and τ > 0 being a decay parameter.

In addition to the weighted input, the digraphs also encompass the directionality of interactions with edge directions determined by electronegativity differences. Capturing directionality allows the model to incorporate key physical and chemical properties of molecular interactions into the spectral features derived from the directed flag complex. Moreover, it provides deeper insights into the mechanistic aspects of our model’s ability to predict protein–ligand binding affinities with a more nuanced representation of interaction networks.

Spectra are generated in a range of filtration values to capture multiscale topological features (Figure 1D). These properties make our bipartite digraphs well suited for spectral analysis, providing information about the connectivity and flow within the protein–ligand interaction network. Supporting Information (Appendix A) discusses in detail how we prepare the input data for PDFL to generate spectra. Furthermore, performance assessment and data preprocessing for machine learning algorithms is covered in Supporting Information (Appendix B).

#### 2.1.1 Statistical Features Extraction

To capture the topological characteristics of our protein–ligand interaction networks, we compute a set of descriptive statistics from the spectral eigenvalues, including the minimum nonzero eigenvalue (the Fiedler value for Laplacian matrices), the maximum eigenvalue, and the sum, mean, median, variance, and standard deviation of all positive eigenvalues. Furthermore, we also incorporate the count of positive eigenvalues, the sum of squares, and the zero count, which represents the number of eigenvalues nearly equal to zero. The analysis is performed over the set of filtration intervals: (0, 0.8), (0.8, 0.85), (0.85, 0.9), (0.9, 0.95), and (0.95, 1), chosen to provide a comprehensive view of the evolving complexity of simplices. We find that the key topological features, Betti-0 and Betti-1, appear to be more concentrated in above-defined intervals. In addition, we consider four protein atoms, {C, N, O, S} and nine ligand atoms, {C, N, O, S, P, F, Cl, Br, I}. Hydrogen (H) atom is excluded to reduce complexity. This results in a total of 3600 = 36*[Element-types]*5[Intervals]*2[Betti numbers]*10[Statistical features] for each protein–ligand complex within a cutoff value of 12 Å. The integration of Betti numbers with the statistical values highlights a systematic approach to incorporate topological and spectral properties of protein–ligand interaction graphs across multiple scales. Moreover, this structured combination ensures that our model captures the full complexity of the interaction network in an interpretable manner. A detailed workflow is demonstrated in Figure 1E.

#### 2.1.2 Hyperparameters

For the quantitative prediction of protein–ligand binding affinities, we built our model by integrating the rigidity index of eq 1 with GBDT. As listed in Table 1, various scales are chosen for the values of β and τ to efficiently represent protein–ligand binding interaction strength. Moreover, we refer to the model as  with β = ν when Ω = *L*, the generalized Lorentz kernel, and β = κ when Ω = *E*, the generalized exponential kernel.

**Table 1:** Various Scales of Hyperparameters for PDFL Model^a^

Parameters	Domain of Values
β	{0.5, 1.0, 1.5,···, 6}
τ	{0.5, 1.0, 1.5,···, 6} ∪ {10, 15, 20}

a Here, β = ν for Ω = L, the generalized Lorentz kernel, and β = κ when Ω = E, the generalized exponential kernel.

#### 2.1.3 Data Preparation for Machine Learning

We validate our PDFL-based machine learning model using three benchmark data sets from the Protein Data Bank (PDB) database:^26,27^ PDBbind v2007^11^ PDBbind v2013^28^ and PDBbind v2016^29^ which are widely used to assess the general performance of scoring functions in diverse protein–ligand complexes.^1,3,11,19,28−36^ Details of the three data sets are given in (Table S1). Furthermore, (Table S2) provides the detailed setting of parameters for our machine learning model (GBDT). For further validation, we also employ 5-fold cross-validation to optimize the kernel hyperparameters Ω, β, and τ using the same parameters for consistency and robustness. We notice that minor variations in these hyperparameters have a little to no effect on the overall prediction accuracy.

### 2.2 Standard PDFL Models for Binding Affinity Predictions

We begin by validating the scoring power of our model by employing the PDBbind v2007 core set, consisting of 195 protein–ligand complexes across 65 clusters as our test set. The training data (*N* = 1105) is compiled based on the refined set which contains 1300 complexes excluding the core set (see Table S1). Moreover, we denote  and  as the mean and best Pearson correlation coefficients, respectively, and similarly, the mean and best cross-validation scores are represented by *CV*^*m*^ and *CV*^*b*^. For the generalized Lorentz kernel model , the Pearson correlation coefficient  is achieved with an RMSE of 1.868 kcal/mol, alongside a 5-fold cross-validation score of *CV*^*m*^, *CV*^*b*^ = (0.743, 0.783). Another model, , performs similarly well, with  and an RMSE of 1.903 kcal/mol, while the cross-validation scores are *CV*^*m*^, *CV*^*b*^ = (0.743, 0.768). Furthermore,  also produces strong results, with a Pearson correlation coefficient of  and an RMSE of 1.883 kcal/mol, together with cross-validation scores of *CV*^*m*^, *CV*^*b*^ = (0.745, 0.768). Among the generalized exponential kernel,  manages to reach comparable performance, with  and an RMSE of 1.905 kcal/mol, with cross-validation scores of *CV*^*m*^, *CV*^*b*^ = (0.731, 0.774). Similarly, the another model  shows  and an RMSE of 1.924 kcal/mol, with cross-validation scores of *CV*^*m*^, *CV*^*b*^ = (0.738, 0.772). Both the models  and  stand very close to , with mean and best Pearson correlation coefficients of 0.823 and 0.827 for both models.  recorded cross-validation scores of 0.736 and 0.773, with an RMSE of 1.890 kcal/mol, while  has slightly higher RMSE at 1.911 kcal/mol, and its cross-validation scores are 0.737 and 0.765. Figure S4 in Supporting Information (Appendix D) illustrated the impact of mean Pearson correlation coefficients (*R*_*p*_) of best performing one-scale  models for the generalized Lorentz kernel and generalized exponential kernel. Among all, , , and  outperform the rest and emerge as the best-performing models with consistently higher correlation coefficient and cross-validation scores (see (Figure S5A)). In contrast, the Lorentz kernel model  with *v* = 15 and τ = 0.5 performs the worst, with , an RMSE of 2.060 kcal/mol, and relatively lower cross-validation scores of *CV*^*m*^, *CV*^*b*^ = (0.694, 0.745). A full comparison of all tested models and results is listed in (Table S3) and their impact is demonstrated in (Figure S7A). Moreover, (Figure S6) represents the mean *R*_*p*_ of the 5-fold cross-validation experiments for the best performing one-scale generalized Lorentz kernel and generalized exponential kernel models, while a combined impact of both kernels can be seen in (Figure S5B).

We further extend our work to incorporate two-kernel models, as they have been shown to improve the accuracy of binding free energy predictions in previous studies,^8,19,25,30,37^ and generate a total of 7200 features against each protein–ligand complex. Among the two-kernel  models tested, the Lorentz–Lorentz configuration  delivers the best performance, with  and an RMSE of 1.865 kcal/mol. Close behind, the mixed Lorentz-exponential model  achieves  and an RMSE of 1.885 kcal/mol. Another mixed kernel model, , shows strong results with , accompanied by an RMSE of 1.885 kcal/mol. Furthermore, the Lorentz-exponential model  and  exhibits Pearson’s correlation values of  and , respectively. The best model among the exponential-exponential configuration is found to be , with an RMSE of 1.906 kcal/mol and . It is evident from these results that the two-scale predictions consistently outperform the single-scale models. Moreover, it becomes apparent that models incorporating generalized Lorentz kernels outperform the models that are only made up of generalized exponential kernels. Finally, the best two-kernel model among the Lorentz–Lorentz configuration is , while the highest score among the exponential-exponential configuration is gained by  ((Figure S7B)).

Based on a comparatively better predictive performance of a two-kernel model than a single-kernel model, we now explore the possibility of utilizing four-kernel PDFL models. In this analysis, a total of 14400 features are extracted for each complex, generated by four corresponding kernels by ensuring that there is no overlap in parameter configuration. This approach allows for a more comprehensive evaluation of multiscale interactions across distinct kernel parameter spaces. Furthermore, we focus on specific sets of β and τ since the entire parametric evaluation is excessively expensive. For the prediction of the PDBbind v2007 core set, feature vectors having zero values for all complexes are ignored which gives rise to a total of 14373 features. We find  to be the best performing model out of all with a mean Pearson correlation of 0.830 and a best value of 0.833 with the RMSE value of 1.873 kcal/mol. Table S4 in Supporting Information (Appendix E) enlists all PDBIDs, experimental, and predicted binding affinities generated by , , and  model on PDBbind v2007 core set.

### 2.3 Consensus PDFL Models for Binding Affinity Predictions

Finally, we develop a consensus PDFL model to further improve the predictive performance by integrating the features generated from our final  model and a pretrained transformer-based predictive model, known to as model-seq. This type of consensus prediction has been performed in a recent study.^38^ Model-seq leverages molecular features using amino acid sequences and SMILES strings data as an input to generate molecular embeddings for proteins^39^ and small molecules.^40^ Then these embeddings are integrated with GBDT algorithm to enhance the predictive accuracy of complex protein–ligand interaction framework. The transformer models yield a total of 1792 features per a protein–ligand pair, with 1280 features from the protein sequence and a total of 512 features from the SMILES representation.

It remains to be shown that the outstanding performance of the PDFL model is not limited to a specific data set. We now consider PDBbind v2013^28^ and train our model on the refined set (*N* = 2959) excluding its core set (*N* = 195) which we use as our test set. With total of 14284 statistical features after the removal of feature vectors with zero values for all complexes, our PDFL-score comes out to be  with an RMSE value of 2.044 kcal/mol. PDBbind v2016 is the last benchmark considered in this study which consists of 290 protein–ligand complexes in 58 clusters.^29^ We train our PDFL model on its refined set (*N* = 4075), excluding its core set. Both training and test data of this data set is larger than its predecessor, PDBbind v2013 with its PDFL-score even better. From a total of 14384 features, the Pearson correlation coefficient ) is achieved with an RMSE of 1.810 kcal/mol. Finally, by integrating the 20 best predictions from all three benchmarks with those from the 20 model-seq predictions into what we refer to as the consensus (PDFL + Transformer) model, we achieve Pearson’s correlation coefficient values of 0.836, 0.808, and 0.851, respectively. In Table 2, mean values of the scoring functions of the PDFL model, Transformer model, and the consensus model (PDFL + Transformer) are provided in the second, third, and fourth column, respectively, together with their corresponding RMSEs.

**Table 2:** Pearson Correlation Coefficients and Root Mean Square Errors (RMSE) (In Kcal/Mol) of the Three Consensus Models^a^

	PDFL	Transformer	PDFL + Transformer
PDBbind-2007	0.830 (1.873)	0.795 (2.006)	0.836 (1.940)
PDBbind-2013	0.770 (2.044)	0.791 (1.977)	0.808 (2.011)
PDBbind-2016	0.819 (1.810)	0.836 (1.716)	0.851 (1.763)

a First column: The three test sets of PDBbind-2007, PDBbind-2013, and PDBbind-2016. Second column: PDFL-Scores along with their corresponding RMSEs. Third column: Transformer-model scores along with the corresponding RMSEs values. Fourth column: The consensus scores of PDFL + Transformer model for the three datasets.

**Figure 2:**
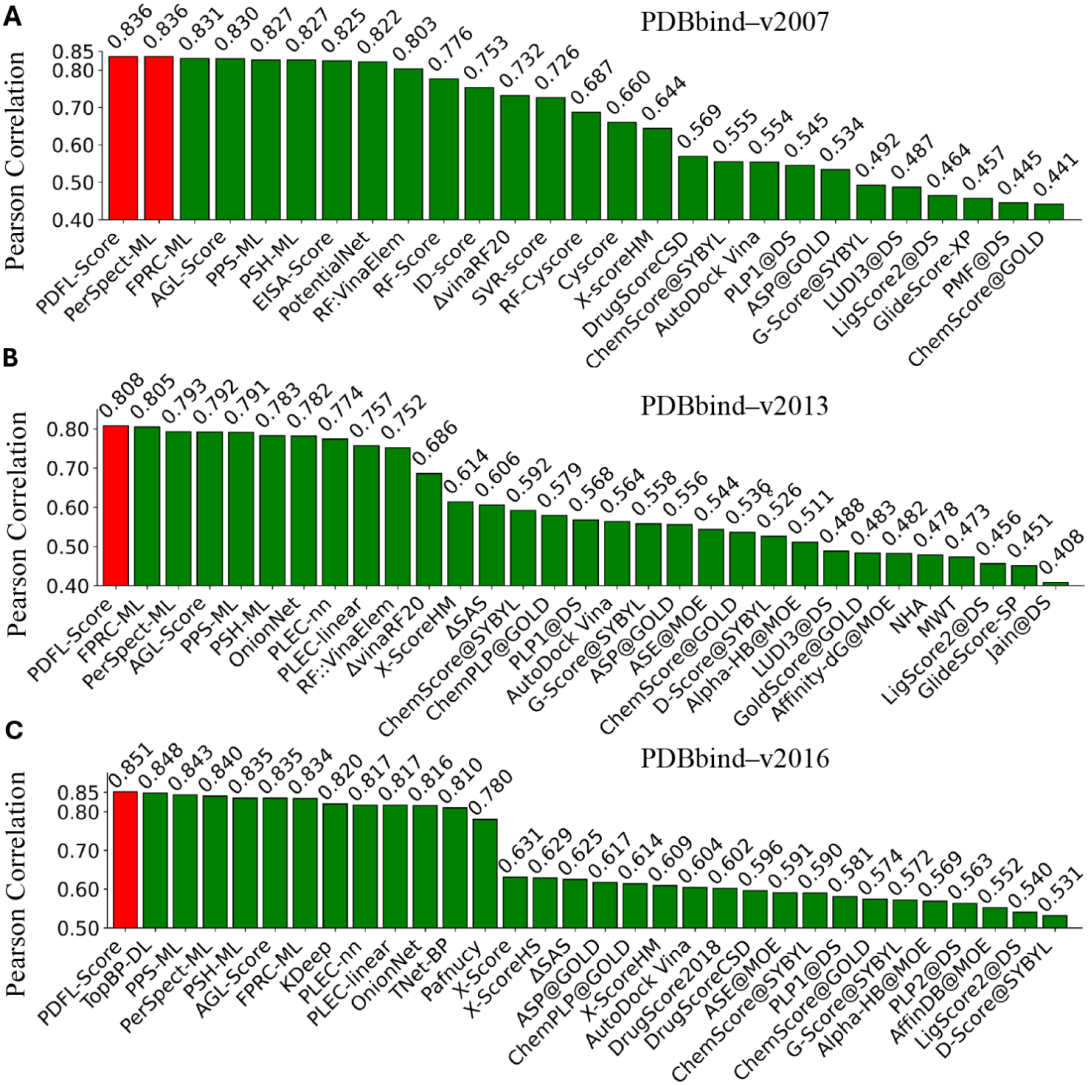
Performance comparison of our PDFL model for three well-established protein–ligand binding affinity data sets, including (A) PDBbind-2007: Our model attains same value of Pearson’s correlation coefficient *R*_*p*_ = 0.836 as the PerSpect-ML model^19^ (both highlighted in red). (B) PDBbind-2013: PDFL-Score outperforms all other methods (highlighted in green), including the FPRC-ML Score with *R*_*p*_ = 0.805^35^ (C) PDBbind-2016: PDFL achieved an *R*_*p*_ = 0.851 (highlighted in red). These results across three data sets validates the robustness and accuracy of the predictive power of PDFL.

In the first benchmark PDBbind-v2007, our PDFL model attains same value of pearson correlation coefficient *R*_*p*_ = 0.836 as the PerSpect-ML model with an RMSE of 1.940 kcal/mol.^19^ The runner-up is the FPRC-ML model^35^ with an *R*_*p*_ of 0.831. Figure 2A shows the performance comparison of the two models along with a number of scoring functions obtained in the earlier studies. As for the second benchmark PDBbind-v2013, the proposed model is able to outperform all the previous scoring functions, including the FPRC-ML^35^ PerSpect-ML^19^ and the AGL^30^ model with an *R*_*p*_ = 0.808 (Figure 2B). The recorded value of root-mean-square error (RMSE) is 2.011 kcal/mol. A full comparison of experimental and predicted binding affinities for multiscale PDFL models for PDBbind-v2013 is presented in (Table S5) along with the corresponding PDBIDs.

**Figure 3:**
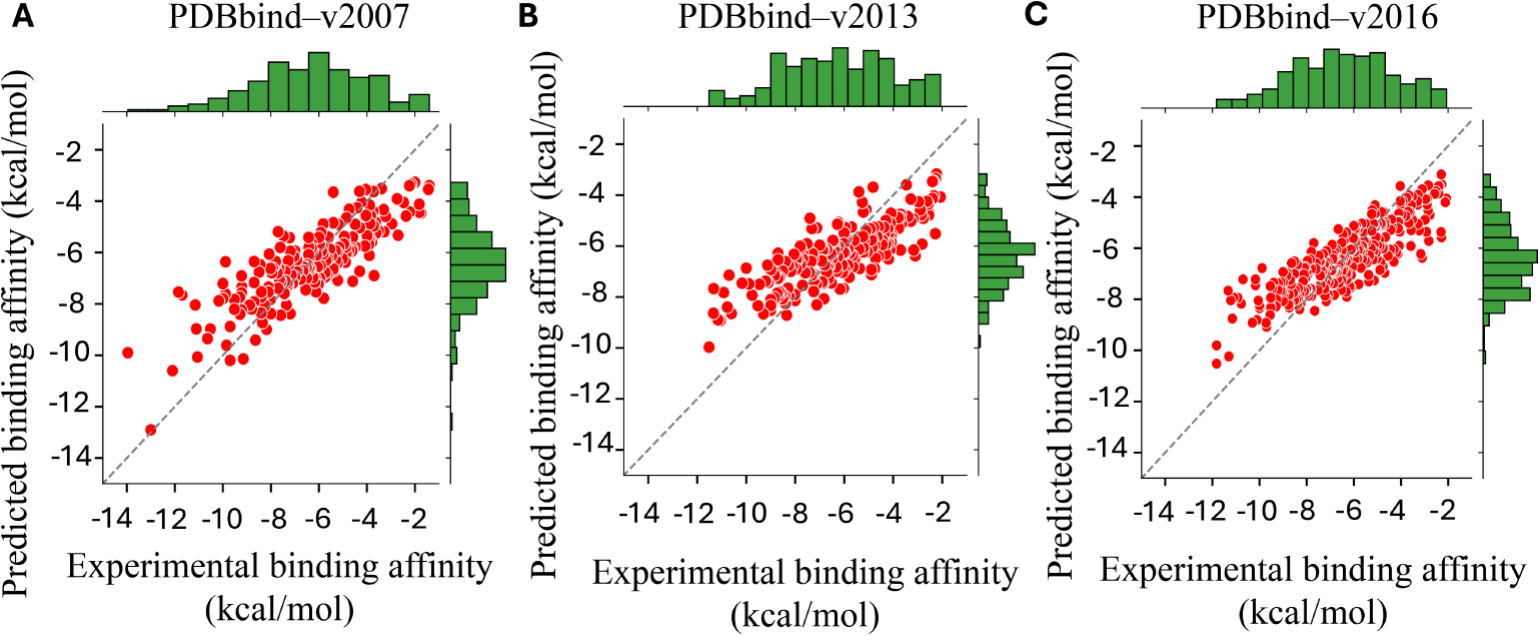
Scatterplot comparison of experimental binding affinities and prediction results for (A) PDBbind-v2007, (B) PDBbind-v2013, and (C) PDBbind-v2016. The corresponding Pearson correlation coefficient values are 0.836, 0.808, and 0.851, respectively.

For PDBbind-v2016, our PDFL model achieves an outstanding *R*_*p*_ value of 0.851 with RMSE = 1.763 kcal/mol. We compare the performance of our PDFL model with various models as shown in Figure 2C. Moreover, a detailed comparison of the experimental and predicted binding affinities generated by , , and  is enlisted in (Table S6). Furthermore, a correlation comparison of experimental binding affinities and prediction results for the three data sets are illustrated in Figure 3. Additionally, a comparative scatterplot analysis to examine the multiscale behavior of the best performing one-scale, two-scale, and four-scale PDFL models for the three data sets are illustrated in (Figures S1–S3, respectively). These results validate the reliability and accuracy of the predictive power of PDFL.

## 3 Methods and Algorithms

**Figure 4:**
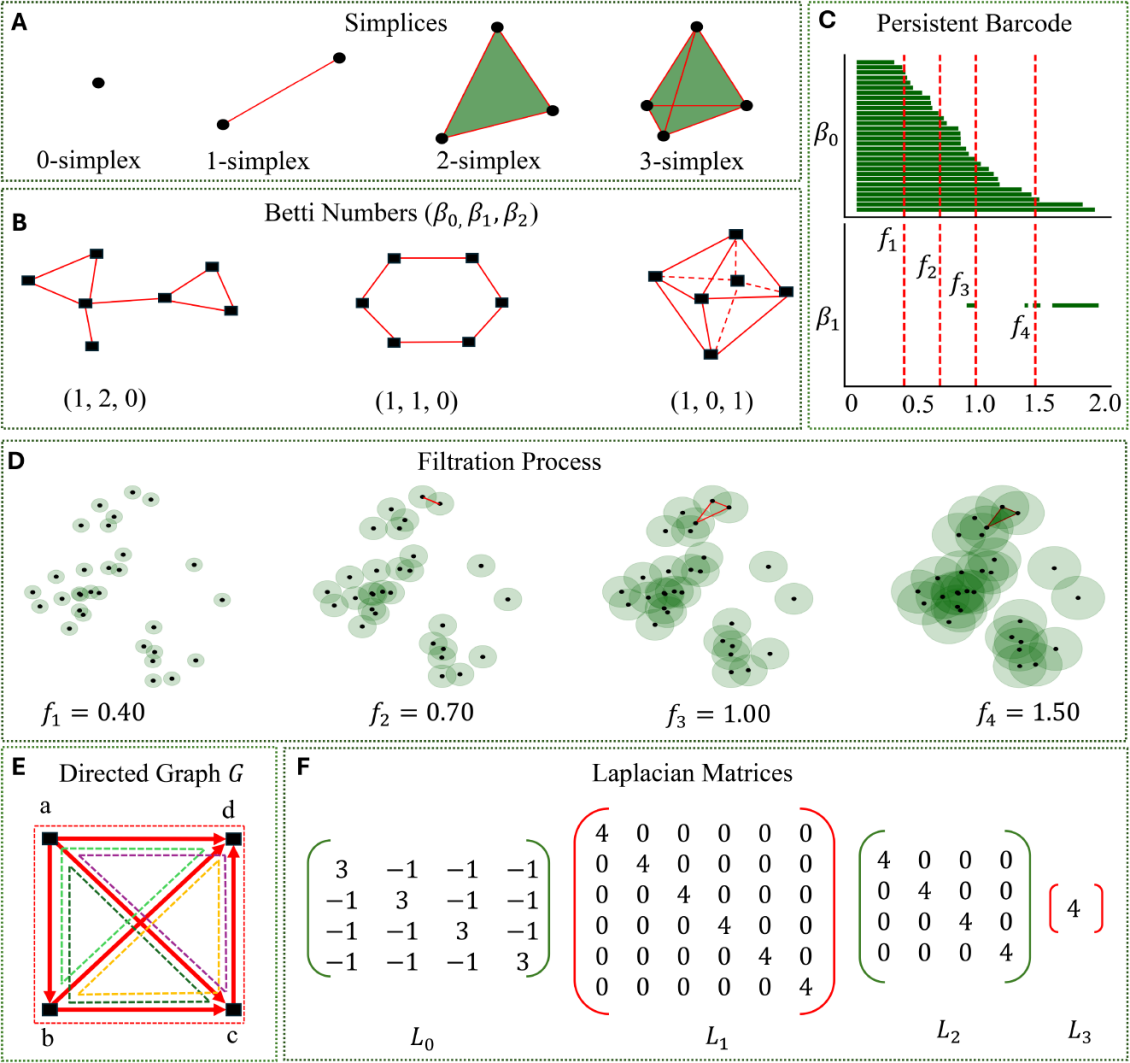
Illustration of fundamental concepts in TDA. (A) A *k*-simplex is made from *k*+1 vertices and can be viewed, geometrically, as a point (0-simplex) defined by exactly 1 vertex, an edge (1-simplex) determined by 2 connected vertices, a triangle (2-simplex) formed by 3 vertices and 3 edges, and a tetrahedron made up of 3-simplex. (**B**) Representation of Betti numbers β_*k*_. Geometrically, β_0_ is the number of connected components, β_1_ is the number of tunnels, loops, or circles, while β_2_ represents the number of voids or cavities. (**C**) Visualization of a persistence barcode rendered from the filtration process. (**D**) Demonstration of a filtration process where simplical complexes are formed by interactions at four distinct filtration values denoted by scales *f*_1_, *f*_2_, *f*_3_, and *f*_4_. (**E**) The directed graph (digraph) *G*, a minimal example of a directed 4-clique, with 4 vertices *V* = {*a*, *b*, *c*, *d*} (all shown in black), 6 edges *E* = {(*a*, *b*), (*a*, *c*), (*a*, *d*), (*b*, *c*), (*b*, *d*), (*c*, *d*)} (all shown in bold red lines with arrows), 4 3-cliques {(*a*, *b*, *c*), (*a*, *b*, *d*), (*a*, *c*, *d*), (*b*, *c*, *d*)} (shown in four different dotted colored lines), and the 4-clique (*a*, *b*, *c*, *d*) (shown in dotted red lines). (**F**) Generation of Laplacian matrices from the graph and directed flag complex of *G*. The spectra of the corresponding matrices *L*_0_, *L*_1_, *L*_2_, and *L*_3_ are computed as {0, 4, 4, 4}, {4, 4, 4, 4, 4, 4}, {4, 4, 4, 4}, and {4}, respectively, along with the Betti numbers β_0_ = 1, β_1_ = 0, β_2_ = 0, and β_3_ = 0.

One of the basic topological representations includes combinatorial structures called simplicial complexes, which are made up simplices and used as a generalization of graphs or networks. Mathematically, a *k*-simplex σ^*k*^ = {*w*_0_, *w*_1_, ..., *w*_*k*_} is the convex hull of affinely independent points , defined as follows:
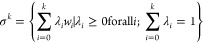


Here, the convex hull formed by any subset of *j* + 1 vertices from the *k* + 1 points {*w*_0_, *w*_1_, ..., *w*_*k*_} is called a *j*-dimensional face of σ^*k*^, where *j* ranges from 0 to *k* – 1. It can be geometrically viewed as a point (0-simplex, 0-clique, and a 0-dimensional face) defined by exactly 1 vertex, an edge (1-simplex, 1-clique, and a 1-dimensional face) determined by 2 connected vertices, a triangle (2-simplex, 3-clique, and a 2-dimensional face) formed by 3 vertices and 3 edges, a tetrahedron made up of (3-simplex, 4-clique, and a 3-dimensional face) defined by 4 vertexes, 6 edges, and 4 triangular faces and so on (Figure 4A).

Homology provides a way to study the topological properties of a space by associating algebraic structures with its geometric features, using tools such as chain groups and boundary operators. *C*_*k*_ is the formal span over *k*-simplices, so *C*_0_ is a vector space with vertices as its basis, *C*_1_ with edges as its basis, and so on. Moreover, for a *k*-simplex, the boundary operator *∂*_*k*_: *C*_*k*_ → *C*_*k*-1_ maps each *k*-simplex to the alternating formal sum of its (*k*-1)-dimensional faces, and is defined as follows:
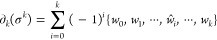
2where {*w*_0_, *w*_1_, ···, *Ŵ*_*i*_, ···, *w*_*k*_} represents the (*k*-1)-simplex obtained by excluding the *i*-th vertex of the *k*-simplex. This forms a chain complex:
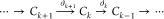


The *k*-cycle group and the *k*-boundary group are given by the kernel of *∂*_*k*_ and the image of *∂*_*k*+1_, respectively: ker(*∂*_*k*_) = {σ ∈ *C*_*k*_|*∂*_*k*_ σ = 0} and im(*∂*_*k*+1_) = {*∂*_*k*+1_ σ|σ ∈ *C*_*k*+1_}. Since *∂*_*k*_ ◦ *∂*_*k*+1_ = 0, we have im(*∂*_*k*+1_) ⊆ ker(*∂*_*k*_), and thus the *k*-th homology group *H*_*k*_ is defined as the quotient of these two groups:
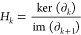


The rank of *H*_*k*_, known as the *k*-th Betti number β_*k*_, indicates the number of independent *k*-dimensional features, such as connected components, cycles, or voids, that are not boundaries of higher-dimensional elements.

This framework allows for systematic identification and classification of topological features, quantified by Betti numbers, and is widely used in molecular representations on multiple scales, within a given configuration,^9,10,41^ In Figure 4B, β_0_ represents the number of connected components, β_1_ is the number of tunnels, loops, or circles, and β_2_ is the number of voids or cavities. At the heart of TDA is persistent homology (PH) which monitors the evolution of these topological features through a process called filtration (Figure 4D). Filtration introduces a parametrization to homology, allowing the analysis of topological features as a parameter-typically related to spatial scale or distance-is varied. This process involves creating a sequence of nested subcomplexes *S*_*i*_, each representing the state of the molecular system at a different spatial resolution for a simplicial complex *S* as follows:
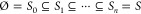


Moreover, a visual representation of these features through persistence barcodes^42^ provides an intuitive way to further comprehend the structure of the given simplicial complex.^43^ The starting point of a bar in a barcode representation indicates the “birth” of a feature, its first appearance during the filtration process, whereas the end point indicates the “death” of a feature, expressing either its disappearance or merging with another feature. In homological terms, the “disappearance” of a feature occurs when it becomes the boundary of a higher-dimensional chain. The length of a bar signifies the “persistence” of the feature. It should be noted here that short-range interactions are characterized by simplicial complexes generated at small filtration values, whereas complexes with larger filtration values represent long-range interactions (Figure 4C).

Various types of simplicial complexes include Vietoris-Rips (VR) complexes, alpha complexes, Čech complexes, and clique complexes. In graph theory, a *k*-clique in an undirected graph *G* = (*W*, *E*) is a set of vertices σ = {*w*_0_, *w*_1_, ..., *w*_*k*-1_} such that each pair of vertices (*w*_*i*_, *w*_*j*_) ∈ *E* is adjacent and mutually connected by an edge without concern for direction. Here, *E* is the set of unordered pairs of vertices representing the edges with a symmetric connection between any two vertices. A clique complex is a simplicial complex where each *k*-clique corresponds to a (*k*-1)-dimensional simplex. For example, a 2-clique (triangle) with 2 vertices {*w*_0_, *w*_1_} corresponds to a 1-simplex in the simplicial complex. For a directed graph *G* = (*W*, *E*), a *k*-clique is an ordered subset of vertices σ = (*w*_0_, *w*_1_, ..., *w*_*k*-1_) such that for *i* < *j*, we have (*w*_*i*_, *w*_*j*_) ∈ *E* and *E* consists of directed edges between the vertices having an asymmetric connection. However, not every set of directed edges between three vertices forms a directed 2-clique. For example, a graph with vertices {*w*_0_, *w*_1_, *w*_2_} and directed edges (*w*_0_, *w*_1_), (*w*_1_, *w*_2_), (*w*_2_, *w*_0_) does not form a directed 2-clique because the edge (*w*_0_, *w*_2_) is missing. For a 2-clique to exist, all necessary directed edges between the vertices must be present. This configuration corresponds to a 3-simplex. The set of all cliques in a directed graph is called a directed clique complex, commonly known as a directed flag complex.

Within the framework of directed graphs (digraphs), a directed flag complex *d F l*(*G*) extends the concept of flag complexes by representing the structure of interactions based on the directionality of edges. In *d F l*(*G*), *k*-simplices are entirely composed of *k*+1 ordered vertices *W*, where the directed edges *E* exist between each consecutive pair of vertices following the order. Moreover, every directed flag complex is an ordered simplicial complex; however, not all ordered simplicial complexes are directed flag complexes because every *k*+1-clique must be included to form a directed flag complex. This means that ordered simplicial complexes that are not directed flag complexes can be generated by the excluding cliques with more than two vertices, i.e., triangles or higher-order cliques.^24^ Persistent Betti numbers provide limited information about the geometric and topological evolution of data. To capture these changes more comprehensively, persistent topological Laplacians offer a deeper analysis. The persistent path Laplacian^17^ bridges the spectral geometry and topological persistence by tracking the shape and structure of directed graphs through a filtration process. It creates a sequence of Laplacian matrices that reflect changes at various filtration levels. Extending this framework, the topological hyperdigraph Laplacian^44^ applies to hypergraphs, which represent complex, asymmetric interactions. This Laplacian captures both harmonic and nonharmonic spectra, paired with persistent homology to monitor the topological evolution of structured data over multiple scales. Lastly, the persistent sheaf Laplacian^45^ unifies the spectral theory with cellular sheaves, encoding both geometric and nongeometric characteristics of point clouds. This technique facilitates the amalgamation of diverse data types, delivering richer insights into the foundational structure of the data.

PDFL extends the concept of combinatorial Laplacian to the setting of a persistent directed flag complex, allowing for a more intricate analysis of spectral properties across various filtration levels.^24^ While directed flag Laplacian serves as a significant tool for analyzing the spectral properties of directed simplicial complexes, it does not capture topological features at different scales in the context of real-world applications. This is where PDFL plays its part by extending the directed flag Laplacian through the incorporation of the concept of persistence, which tracks the evolution of topological features across various filtration levels. In particular, PDFL refines the same combinatorial framework of the directed flag Laplacian with an additional layer of persistence, where there is a filtration of directed flag complexes dFl(*G*_0_) ⊂ dFl(*G*_1_) ⊂ ···. This enables the study of topological features on multiple scales. In addition, the chain groups (or vector spaces) *C*_*k*_ are defined with coefficients in  and endowed with an inner product. This structure introduces a persistent boundary operator  associated with filtration levels *a* and *b* for each *k* ≥ 0 and *a* ≤ *b*, along with its adjoint , induced by the structure of the inner product. These elements combine to form the *k*-th (*a*, *b*)-persistent directed flag Laplacian , defined as follows:
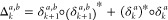
3

with the persistent boundary operator defined as
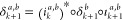
4

Here,  is expressed in terms of the inclusion morphisms  and , where . This means that  consists of chains in  whose boundaries lie entirely within . These inclusion morphisms are crucial in the construction of persistent homological structures and allow us to relate chain complexes at various filtration levels. In addition, the adjoint of the boundary operators and inclusion morphisms follow a similar structure. They play a fundamental role in ensuring that the Laplacian is positive semidefinite and self-adjoint by preserving the inner product structure of the chain groups. With  and  representing the boundary matrices corresponding to  and , respectively, the matrix representation of the positive semidefinite persistent Laplacian  is given by
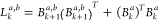
5

The spectrum , arranged in nondecreasing order, consists of real and non-negative eigenvalues. These eigenvalues are crucial for understanding the spectral properties of the complex and tracking how persistent topological features develop across various filtration scales. In particular, the smallest nonzero eigenvalue of  is especially significant and is denoted by . A minimal example of a directed 4-clique, denoted by *G*, is presented in Figure 4E. The spectra of the corresponding Laplacian matrices *L*_0_, *L*_1_, *L*_2_, and *L*_3_ is calculated (Figure 4F) which results in Betti numbers β_0_ = 1, β_1_ = 0, β_2_ = 0, and β_3_ = 0, respectively.

## 4 Conclusion

Persistent homology (PH) and persistent Laplacian (PL) are well-established strategies in topological data analysis (TDA) for analyzing biomolecular systems. However, these methods overlook factors such as polarization, heterogeneity, and directed behavior in chemical, physical, and biological interactions. To address these limitations, we introduce the persistent directed flag Laplacian (PDFL) model as a novel approach for modeling directed interactions and apply it to predict protein–ligand binding affinities. In addition to the confirmed predictive accuracy and reliability of our PDFL model, it delivers simplicity by expecting only raw inputs, without the need for complex data optimization or processing. We simplify data processing by incorporating a detailed network of digraphs into our model with the correlation matrix functioning as the foundation for interaction weights and electronegativity facilitating the edge directions. This simple construction enables us to preserve the accuracy by highlighting the fundamental characteristics to represent interactions between atoms.

Using the fast algorithmic power of the PDFL tool, this multiscale framework generates the underlying topological structures of the system in the form of spectra in seconds. This asserts its position as a powerful tool and vital asset in biomolecular modeling and drug discovery. Moreover, the proposed study marks the first real-world application of PDFL and introduces the first topological modeling of polarized interactions in molecular science. The success of PDFL demonstrates its high applicability not only to biomolecular modeling, directed evolution, protein engineering, and drug discovery but also to data science more broadly.

## Data Availability

All codes needed to evaluate the conclusions in this study are available at https://github.com/mzia-s/PDFL-Score, while the source codes for the PDFL tool can be found at https://github.com/bdjones13/flagser-laplacian/. The PDBbind data sets, can be downloaded from https://www.pdbbind.org.cn.

## Supporting Information Available

Detailed computational methods, benchmark data sets used in this study (Table S1), data preparation procedures, model validation details, and hyperparameter settings (Table S2). Model prediction correlations and validation analyses (Figures S1–S3), hyperparameter performance evaluation (Figures S4–S7), PDFL-based binding affinity predictions for the single-scale model (Table S3), and comprehensive comparisons of experimental versus predicted binding affinities across benchmark data sets (Tables S4–S6) (PDF)
