# Supplementary material for: Persistent Directed Flag Laplacian (PDFL)-Based Machine Learning for Protein–Ligand Binding Affinity Prediction
Source: J Chem Theory Comput. 2025 Apr 5;21(8):4276–85. doi: 10.1021/acs.jctc.5c00074 (PMC12020371; doi:10.1021/acs.jctc.5c00074)
Supplement: Supplementary file 1 — ct5c00074_si_001.pdf [file ct5c00074_si_001.pdf]

Supporting Information

Persistent Directed Flag Laplacian (PDFL)-Based Machine Learning for Protein–Ligand Binding Affinity Prediction

Mushal Zia[1], Benjamin Jones[1], Hongsong Feng[1], and Guo-Wei Wei[1,2,3,†]
[1] Department of Mathematics,
Michigan State University, East Lansing, MI 48824, USA.
[2] Department of Electrical and Computer Engineering,
Michigan State University, East Lansing, MI 48824, USA.
[3] Department of Biochemistry and Molecular Biology,
Michigan State University, East Lansing, MI 48824, USA.
[†] Address correspondences to Guo-Wei Wei. E-mail: weig@msu.edu

APPENDIX A

COMPUTATIONAL IMPLEMENTATION OF PDFL

A1. Correlation Functions and Protein-Ligand Analysis

Persistent homology (PH) unveils the fundamental connectivity within biomolecular complexes and enables accurate predictions of protein-ligand binding affinity. This is done by abstracting intricate topological features and reducing the dimensionality. Topological fingerprints (TFs) [1, 2] play a key role in this framework by providing a detailed characterization of the spatial and interaction patterns within biomolecular complexes. These TFs encompass various topological invariants and are typically visualized using barcodes [3] or persistence diagrams. Although TFs can capture connectivity patterns as the filtration parameter varies and identify persistent topological structures, they often overlook critical atomic-level details. To tackle this constraint, we employ element-specific topological fingerprints (ES-TFs) [4], which take into account the distinct elemental composition of proteins and ligands, facilitating a more refined characterization of biomolecular interactions. We represent these interactions by topological connections between atom pairs, denoted as (P - L), where P refers to a protein atom and L refers to a ligand atom. This notation captures the general connectivity between the two entities and provides a biologically relevant analysis of binding interactions. For example, a carbon-oxygen (C - O) graph represents interactions by linking the carbon atom in the protein with the oxygen atom in the ligand.

When studying the 3D structure of biomolecules, such as proteins, we consider N atoms, each represented by their position as $\{\mathbf{x}_i \mid \mathbf{x}_i \in \mathbb{R}^3, i = 1, 2, \ldots, N\}$. The Euclidean distance between any i-th and j-th atom is denoted by $||\mathbf{x}_i - \mathbf{x}_j||$. For the quantification of the interaction between atoms based on this distance, we introduce the characteristic distance $\eta_{ij} = \tau(r_i + r_j)$, where r_i and r_j represent the van der Waals radii of atoms i and j, respectively, and $\tau > 0$ controls the decay rate of the interaction strength, efficiently tuning the spatial scale at which the atomic interactions are significant. Alongside the topological perspective, it is crucial to quantify atomic interaction strengths beyond relying solely on Euclidean distances. A more refined approach uses decaying radial basis functions, often referred to as kernels, to transform these distances into interaction measures.

The flexibility-rigidity index (FRI), introduced in earlier studies [5–7], offers an effective computational framework to predict B factors and evaluate the atomic-level flexibility based on atomic coordinates. The FRI formulation incorporates a correlation function, denoted as Φ, which maps Euclidean distances between atoms to values that signify the strength of their pairwise interactions. The rigidity index μ_i and the flexibility index f_i of atom i are calculated based on a weighted sum of interactions with all other atoms, using the following expressions:

$$\mu_i = \sum_{j=1}^{N} \omega_j \Phi(||\mathbf{x}_i - \mathbf{x}_j||; \eta_{ij}), \tag{1}$$

$$f_i = \frac{1}{\mu_i}. \tag{2}$$

Here, ω_j are the element-specific weights, which are set to 1 for all atoms to reduce the number of free parameters. The function Φ is a real-valued, monotonically decreasing correlation function that describes how interactions between atoms diminish with increasing distance. It depends on the Euclidean distance $||\mathbf{x}_i - \mathbf{x}_j||$ and the characteristic distance η_{ij}. The behavior of Φ is governed by the following properties [6]:

$$\Phi(||\mathbf{x}_i - \mathbf{x}_j||; \eta_{ij}) = \begin{cases} 1, & \text{as } ||\mathbf{x}_i - \mathbf{x}_j|| \to 0 \\ 0, & \text{as } ||\mathbf{x}_i - \mathbf{x}_j|| \to \infty \end{cases} \tag{3}$$

The most commonly used FRI correlation functions include generalized exponential functions defined as

$$\Phi^{\exp}_{\kappa,\tau}(||\mathbf{x}_i - \mathbf{x}_j||;\eta_{ij}) = e^{-\left(\frac{||\mathbf{x}_i-\mathbf{x}_j||}{\eta_{ij}}\right)^{\kappa}}, \quad \kappa > 0 \tag{4}$$

and generalized Lorentz functions defined as

$$\Phi^{\mathrm{lor}}_{\nu,\tau}(||\mathbf{x}_i - \mathbf{x}_j||;\eta_{ij}) = \frac{1}{1+\left(\frac{||\mathbf{x}_i-\mathbf{x}_j||}{\eta_{ij}}\right)^{\nu}}, \quad \nu > 0 \tag{5}$$

The exponential kernel decays rapidly, and the parameter κ controls the rate of this decay. The Lorentz kernel decays more slowly, making it suitable for long-range interactions, with the parameter ν adjusting the sharpness of the decay.

In the present work, we construct an element-level multiscale weighted rigidity index that captures the total interaction strength between protein (P) and ligand (L) pairs. This strength of interaction is quantified by summing up the contributions from all pairs of protein atoms $p \in P$ and ligand atoms $l \in L$, as expressed in the following rigidity index equation:

$$\begin{aligned} RI^{\Omega}_{\beta,\tau,c}(P-L) = \sum_{p\in P} \mu^{\Omega}_p = \sum_{p\in P}\sum_{l\in L} \Phi^{\Omega}_{\beta,\tau}(||\mathbf{r}_p - \mathbf{r}_l||;\eta_{pl}), \\ \forall\ ||\mathbf{r}_p - \mathbf{r}_l|| \leq c \end{aligned} \tag{6}$$

where $\Omega = E$ or $\Omega = L$ refers to a kernel index. The term μ^{Ω}_p represents the rigidity index of a given protein atom p, which is the sum of its interactions with all ligand atoms $l \in L$. The total rigidity index $RI_{\beta,\tau}(P-L)$ sums over all protein atoms to provide a global measure of rigidity for the entire protein-ligand interaction network within a predefined cutoff distance c, thus reducing computational complexity.

A2. Electronegativity-Based Edge Directionality

The proposed multiscale system given in Equation (6) represents a bipartite directed graph, as the interactions (edges) are directed exclusively between protein atoms and ligand atoms, with no interactions within the same group. As a result, this work exclusively focuses on the 0-dimensional Laplacian. In (6), protein atoms P and ligand atoms L form two distinct sets, and the edges between them are weighted using a transformed correlation matrix. These edges represent the spatial interaction and connectivity between a protein atom p and a ligand atom l, defined as $1-\kappa(p,l)$, where $\kappa(p,l) = \Phi^{\Omega}_{\beta,\tau}$. The resulting transformed values connect the nodes representing a protein atom p to a ligand atom l. This transformation yields values in the range $(0,1]$ and ensures that shorter distances between protein-ligand pairs result in higher interaction strengths, while more distant pairs result in weaker interactions. Furthermore, the directionality of the edges in this graph is determined on the basis of the electronegativity values χ_p and χ_l of the protein and ligand atoms. We set an edge directed from p to l for $\chi_p < \chi_l$, representing a tendency of the electron density to flow toward the more electronegative ligand atom. Conversely, if $\chi_p > \chi_l$, the edge is directed from l to p. A bonding environment is taken into account when both atoms share the same value of electronegativity. As an example, we consider the case of a weighted edge $(N-N)$ where both the protein and the ligand nitrogen atoms have the same electronegativity value of 3.04. We then examine the bond limit of 1.55 Å between the nitrogen protein atom S_p and the nitrogen ligand atom S_l to determine a close proximity of forming a bond. If bonded, we determine the sum of the electronegativity values for the atoms bonded to S_p and S_l. As a result, an edge is directed from the protein N atom to the ligand N atom if $S_p < S_l$; otherwise, the edge is directed from the ligand N atom to the protein N atom (i.e., for $S_l < S_p$). With the correlation matrix functioning as the foundation for interaction weights and electronegativity facilitating the edge directions, this simple construction enables us to record both the directionality and strength of interactions between protein and ligand atoms.

A3. The Persistent Directed Flag Laplacian (PDFL) Tool

The PDFL tool requires a simple input structured in flagser format [8, 9]. The tool needs only a few atomic details, such as names, radii, bond lengths, electronegativity values, and the corresponding 3D coordinates, which can easily be obtained from the PDBbind molecular database. Protein atoms are filtered within a proximity of 12Å and the interaction strength between protein and ligand atoms is determined using generalized Lorentz or generalized exponential functions. Next, a directed graph is constructed from the linkage between protein and ligand atoms, where atoms serve as nodes and directed edges, weighted by the kernel function, are formed based on the electronegativity difference. The filtration parameter in PDFL varies independently from these edge weights. In this study, we specify a filtration range from 0 to 1, incremented by 0.01, and at each filtration level ε, PDFL computes the spectra of Laplacian matrices for the subgraph corresponding to edges with filtration values below ε.

APPENDIX B

DATA PREPARATION AND MODEL EVALUATION METRICS

B1. Datasets

Scoring functions typically fall into four categories [10]: (a) Force-field-based models, which use physical interactions such as van der Waals forces, electrostatics, and hydrogen bonding to explain binding mechanisms. (b) Empirical models that treat the binding affinity as a sum of interaction types and factors like desolvation. (c) Knowledge-based models that derive potentials from large datasets of known complexes. (d) Machine learning models, which use advanced algorithms and large datasets to predict binding affinity with greater flexibility and accuracy. Our approach falls into the category of machine learning. In this work, different PDFL models are generated and machine learning predictions are obtained on different training and testing datasets. Protein Data Bank (PDB) has a comprehensive collection of protein-ligand complexes with experimentally determined atomic structures and binding affinities, which makes it a necessary resource for the robust evaluation of scoring functions used in protein-ligand binding affinity prediction, molecular interactions, and drug discovery. We used three widely used datasets, namely PDBbind-v2007, PDBbind-v2013, and PDBbind-v2016. The details of the training and testing sets of the three datsets are given in Table S1.

Table S1: Details of the three PDBbind databases.

Version	Refined Set	Training Set	Test Set (Core Set)
v2007	1300	1105	195
v2013	2959	2764	195
v2016	4057	3772	285

B2. Pearson's Correlation Coefficient (R_p)

We employ scoring power as a key evaluation metric to validate our model's ability to predict protein-ligand binding affinities. For normally distributed variables X and Y, Pearson's correlation coefficient (PCC) measures the strength and direction of the linear relationship between them and is often denoted as R_p:

$$R_p = \text{PCC}(X,Y) = \frac{\sum(x_i - \bar{x})(y_i - \bar{y})}{\sqrt{\sum(x_i - \bar{x})^2}\sqrt{\sum(y_i - \bar{y})^2}} \tag{7}$$

Here, x_i and y_i represent the predicted and experimental binding affinities for the i-th complex, respectively, while $\bar{x}$ and $\bar{y}$ denote the mean values of the predicted and experimental affinities. A higher value of R_p close to 1 indicates a stronger linear correlation between the predicted and experimental affinities.

B3. Root Mean Square Error (RMSE)

Another commonly used evaluation metric is the root mean squared error *RMSE* which we have utilized in this study to evaluate the effectiveness of our machine learning regression model and is defined as:

$$RMSE = \sqrt{\frac{1}{n}\sum_{i=1}^{n}(y_i - \hat{y}_i)^2} \tag{8}$$

where y_i and $\hat{y}_i$ are the true and predicted values of the i-th complex, respectively, and n is the total number of samples. The metrics, R_p and *RMSE*, provides a valuable insight into the predictive performance of a model under observation. It is noted that the binding energy unit is pK_d in the PDBbind database and is multiplied by a constant value of 1.3633 for conversion into kcal/mol unit.

B4. GBDT Fine-Tuning

During the course of this study, we use a machine learning model, the gradient boost decision tree (GBDT), to build regression models, from the Scikit-learn [11] v1.5.1 package, for its ability to optimize loss functions and handle both linear and non-linear data. The model is configured with 20,000 estimators, a maximum depth of 8, a minimum of 6 samples per split, a learning rate of 0.005, and a subsampling ratio of 0.7. We apply the squared error loss function, use the square root of all the features in each split, and ensure consistent randomization by repeating each experiment 20 times with different random seed (Table S2). For further validation, we employ 5-fold cross-validation to optimize the kernel hyperparameters Ω, β, and τ using the same parameter values for consistency. Minor variations in these hyperparameters have minimal effect on the overall prediction accuracy.

Table S2: The setting of parameters for our GBT model.

**No. of estimators**	**Maximum depth**	**Minimum sample split**	**Learning rate**
20,000	8	6	0.005
**Loss function**	**Maximum features**	**Subsample size**	**Repetition**
Least square	Square root	0.7	20 times

APPENDIX C

MULTI-SCALE ANALYSIS OF PDFL MODELS IN BINDING PREDICTION

C1. Scatterplot Comparisons Across PDBbind Datasets

This section presents a comparative scatterplot analysis to examine the multi-scale behavior of PDFL models using training data from PDBbind-v2007, PDBbind-v2013, and PDBbind-v2016, respectively.

We evaluate our best-performing one-scale model, $\mathrm{PDFL}^{L}_{3,2}$, alongside the highest-performing two-scale Lorentz model, $\mathrm{PDFL}^{LL}_{3,2;3,1}$, and the four-scale consensus model, $\mathrm{PDFL}^{LLEE}_{3,2;3,1;10,3.5;6,2.5}$.

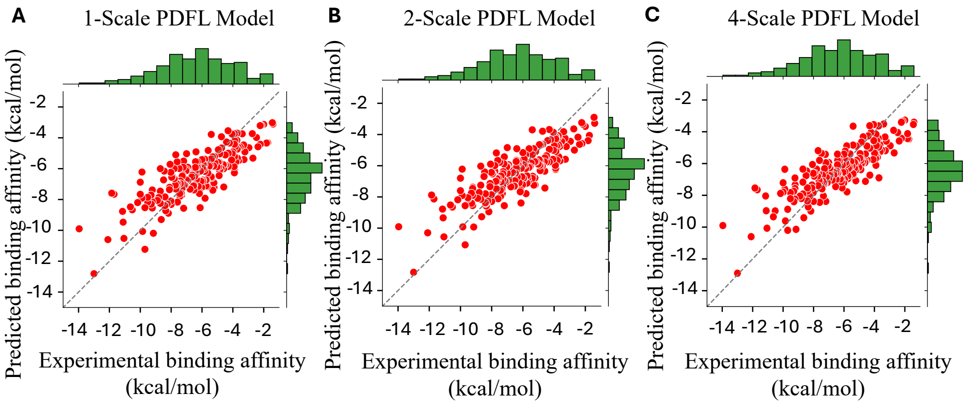

Figure S1: PDBbind-v2007: Mean R_p values for 1-scale, 2-scale, and 4-scale PDFL models are 0.828, 0.830, and 0.836, respectively, with corresponding RMSE values (kcal/mol) of 1.868, 1.865, and 1.940.

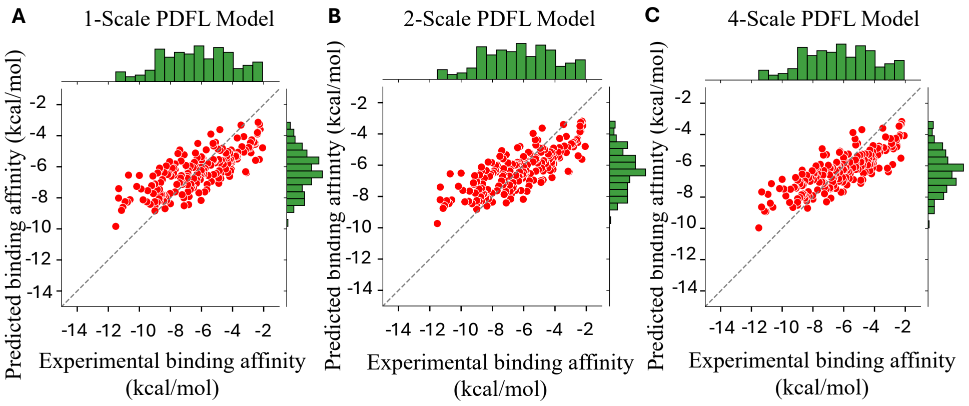

Figure S2: PDBbind-v2013: Mean R_p values for 1-scale, 2-scale, and 4-scale PDFL models are 0.760, 0.770, and 0.808, respectively, with corresponding RMSE values (kcal/mol) of 2.053, 2.040, and 2.011.

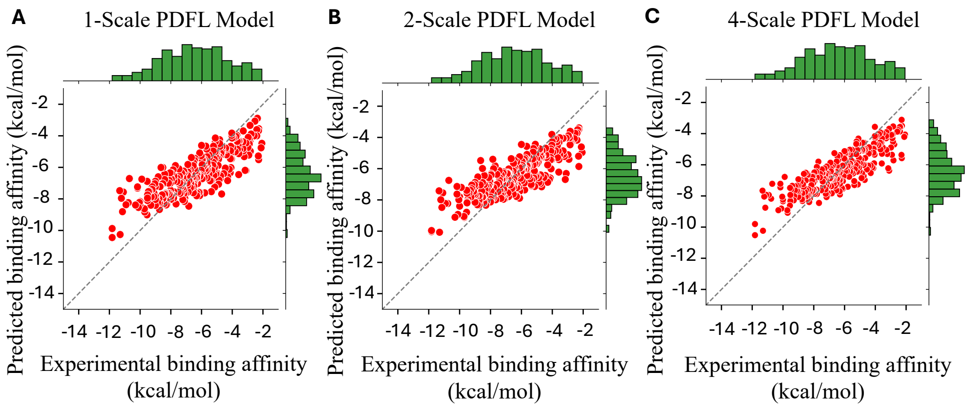

Figure S3: PDBbind-v2016: Mean R_p values for 1-scale, 2-scale, and 4-scale PDFL models are 0.824, 0.821, and 0.851, respectively, with corresponding RMSE values (kcal/mol) of 1.773, 1.813, and 1.763.

APPENDIX D

ADDITIONAL RESULTS FOR PDBBIND-V2007

D1. One-Scale and Two-Scale Behavior of PDFL Models in Protein-Ligand Binding Prediction

This section highlights the multiscale behavior of PDFL models in protein-ligand binding predictions on the training data from the PDBbind-2007 benchmark. Figure S4 to Figure S7 represents the impact of the Pearson's correlation coefficient average and the 5-fold cross-validation (R_p) of one-scale PDFL models plotted against various combinations of τ and κ (or ν). In this work, we have utilized β = {0.5, 1.0, 1.5,..., 6} and τ = {0.5, 1.0, 1.5,..., 6} ∪ {10, 15, 20} as model hyperparameters.

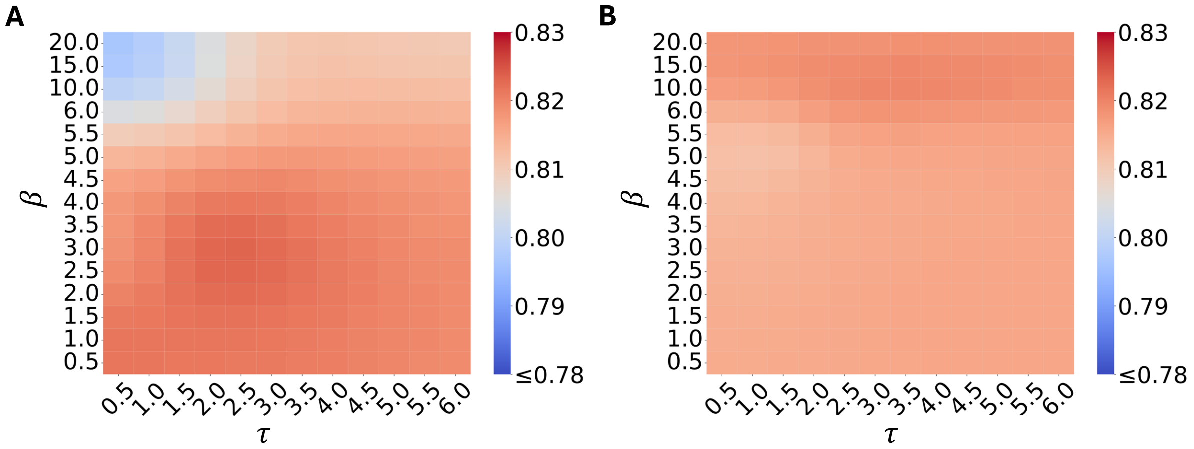

Figure S4: Mean Pearson correlation coefficients (R_p) of various one-scale PDFL$^{\Omega}_{\beta,\tau}$ models. The highest mean value of various one-scale hyperparameters for $\beta = \nu$ when $\Omega = L$ (the generalized Lorentz kernel) and $\beta = \kappa$ when $\Omega = E$ (the generalized exponential kernel) are found to be **(A)** PDFL$^{L}_{3,2}$ = 0.828 and **(B)** PDFL$^{E}_{10,3.5}$ = 0.823, respectively.

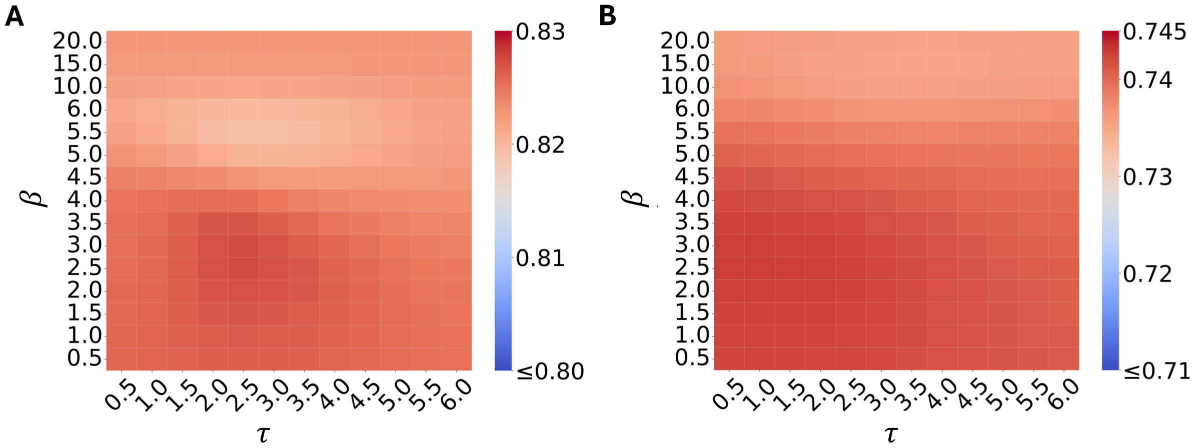

Figure S5: Mean Pearson correlation coefficients (R_p) and 5-Fold Cross validation of best performing one-scale PDFL Model. **(A)** The mean value of best performing one-scale hyperparameters for all generalized Lorentz kernel and the generalized exponential kernels (see Table S3) plotted against the same combination of parametric scales. **(B)** PDFL$^{\Omega}_{\beta,\tau}$ for best generalized Lorentz kernel and the generalized exponential kernels for 5-fold cross validation (see Table S3) plotted against the same combination of parametric scales.

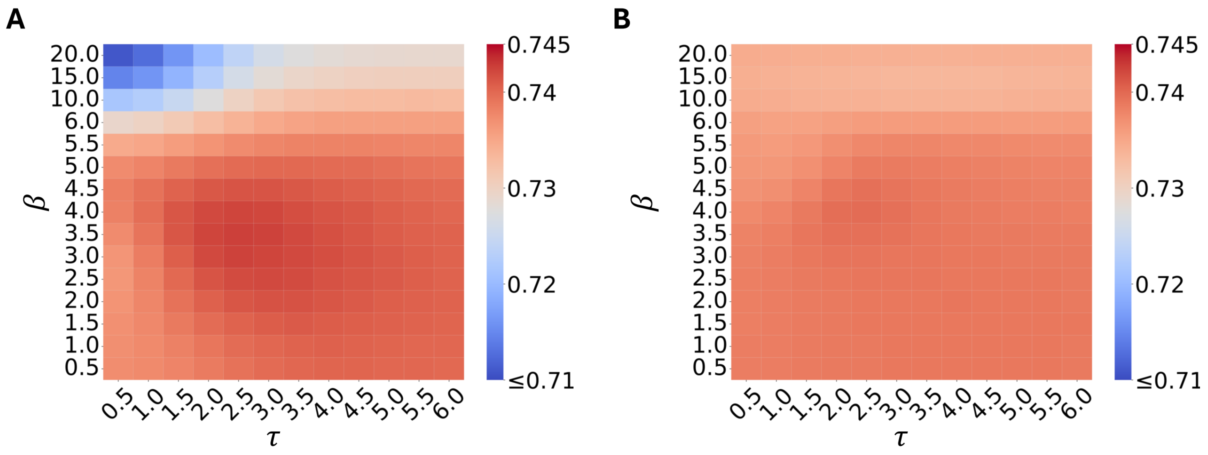

Figure S6: Mean Pearson correlation coefficients (R_p) of the 5-fold cross-validation experiments for various one-scale PDFL$^{\Omega}_{\beta,\tau}$ models. The mean value of best performing one-scale hyperparameters for $\beta = \nu$ when $\Omega = L$ and $\beta = \kappa$ when $\Omega = E$ are respectively found to be **(A)** PDFL$^{L}_{3,2}$ = 0.743 and PDFL$^{L}_{3,1}$ = 0.743, **(B)** PDFL$^{E}_{10,3.5}$ = 0.731 and PDFL$^{E}_{6,2.5}$ = 0.738.

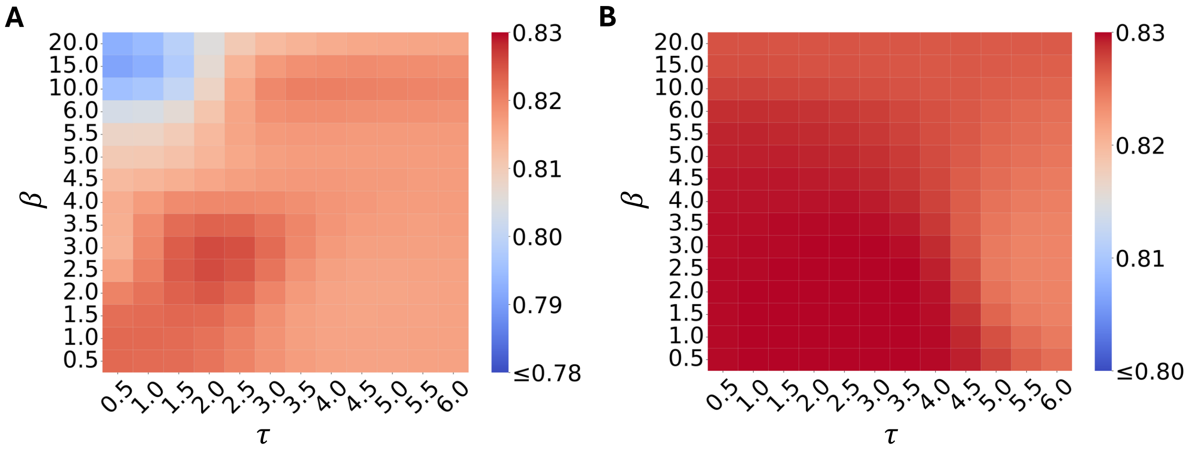

Figure S7: Mean Pearson correlation coefficients (R_p) of the experiments for all one-scale PDFL model and best two-scale PDFL models. **(A)** PDFL$^{\Omega}_{\beta,\tau}$ for all generalized Lorentz kernel and the generalized exponential kernels (see Table S3) plotted against the same combination of parametric scales. **(B)** Highest mean values of best performing two-scale PDFL models, PDFL$^{LL}_{3,2;3,1}$ = 0.830 and PDFL$^{EE}_{10,3.5;6,2.5}$ = 0.824, are plotted against various combinations of τ and κ.

D2. Protein-Ligand Binding Affinity Predictions for Single-Kernel PDFL Model

This section presents the mean and best Pearson correlation coefficient (R_p) values along with their respective root mean square error (RMSE). The unit for RMSE values is kcal/mol, which is converted from kJ/mol by multiplying with the inverse square root of the energy unit conversion factor (4.184) for our PDFL-Machine learning-based GBT model. Moreover, the corresponding mean and best 5-fold CV

scores for the PDBbind-2007 test sets are also mentioned in the third column of Table S3.

Table S3: PDFL-Scores for One-Scale Model. The Pearson's correlation coefficient (R_p) values and Root mean square error (RMSE) (in kcal/mol) for our PDFL-Machine learning based GBT model on the test sets of PDBbind-2007 with its corresponding 5-fold CV scores..

PDFL$^{\Omega}_{\beta,\tau}$	R_p (Mean) [Best]	5-fold CV (Mean) [Best]	RMSE
$L[3,2]$	0.828[0.831]	0.743 [0.783]	1.868
$L[3,1]$	0.825 [0.829]	0.743 [0.768]	1.903
$L[3.5,1.5]$	0.824 [0.827]	0.745 [0.768]	1.883
$E[10,3.5]$	0.823 [0.827]	0.731 [0.774]	1.905
$L[15,3]$	0.823 [0.827]	0.736 [0.773]	1.890
$L[1.5,0.5]$	0.823 [0.828]	0.737 [0.765]	1.911
$E[6,2.5]$	0.818 [0.821]	0.738 [0.772]	1.924
$E[2,4]$	0.816 [0.821]	0.739 [0.764]	1.919
$E[4.5,2]$	0.816 [0.819]	0.741 [0.768]	1.929
$E[5,1.5]$	0.809 [0.814]	0.735 [0.758]	1.957
$L[6,1]$	0.808 [0.812]	0.737 [0.7636]	1.992
$L[3,0.5]$	0.806 [0.811]	0.726 [0.748]	1.997
$L[10,1]$	0.794 [0.798]	0.725 [0.749]	2.083
$L[15,0.5]$	0.7807 [0.783]	0.694 [0.745]	2.060

APPENDIX E

EXPERIMENTAL VS. PREDICTED BINDING AFFINITIES

E1. Results for the PDBbind v2007 Dataset

Table S4 presents the PDBID, experimental binding affinities (Exp BA), and predicted binding affinities (Pred BA) obtained from the PDFL models on the PDBBind v2007 core set. The superscripts a, b, and c in Pred BA denote predictions from the best performing one-scale (PDFL$^{L}_{3,2}$), two-scale (PDFL$^{LL}_{3,2;3,1}$), and four-scale models (PDFL$^{LLEE}_{3,2;3,1;10,3.5;6,2.5}$), respectively.

Table S4: PDBbind v2007: Experimental and Predicted Binding Affinities.

PDBID	Exp BA	Pred BA^a	Pred BA^b	Pred BA^c	PDBID	Exp BA	Pred BA^a	Pred BA^b	Pred BA^c
2hdq	1.4	3.11	3.28	3.4	1utp	1.44	3.02	2.9	3.54
1u2y	1.74	4.32	4.06	4.46	1bra	1.82	4.37	4.42	4.43
1y1m	1.82	4.34	4.39	4.12	3pce	2.0	3.13	3.48	3.27
4tim	2.16	3.2	3.34	3.34	1m0n	2.22	4.42	4.58	4.42
1ajp	2.23	3.21	3.24	3.4	6rnt	2.37	4.58	4.63	4.58
1tok	2.47	3.98	4.17	4.05	2fzc	2.7	5.32	5.0	5.33

PDBID	Exp BA	Pred BA[a]	Pred BA[b]	Pred BA[c]	PDBID	Exp BA	Pred BA[a]	Pred BA[b]	Pred BA[c]
2qwb	2.74	3.73	3.64	3.91	1f4e	2.96	4.64	4.44	4.88
1zc9	3.22	4.91	4.77	4.59	1bcu	3.28	5.41	5.59	5.19
1d7j	3.3	4.46	4.24	4.33	1toj	3.39	3.74	3.82	3.51
1jys	3.52	4.6	4.44	5.15	1pb9	3.62	5.47	5.68	5.25
2azr	3.64	4.64	4.76	4.82	1loq	3.7	5.32	5.43	6.9
1ai5	3.72	3.7	4.17	3.63	4tln	3.72	4.74	4.66	4.96
1f5k	3.74	4.16	4.08	4.41	1nje	3.8	5.56	5.66	5.6
1j16	3.84	4.58	4.74	4.56	1v16	3.87	4.2	4.17	4.87
1m0q	3.89	3.73	4.18	4.16	2ctc	3.89	5.22	5.09	5.42
1avn	3.9	4.42	4.28	5.53	1slg	3.9	6.08	6.24	6.07
2rkm	3.9	6.27	6.16	6.11	1pr5	3.92	5.87	5.98	5.68
2g8r	3.99	4.91	4.73	5.35	2c02	4.04	5.03	5.01	4.56
1toi	4.05	3.76	3.86	3.55	1n2v	4.08	5.44	5.59	5.75
1kv5	4.22	4.34	4.33	4.04	1a30	4.3	5.86	5.75	6.73
1det	4.3	5.0	4.95	5.03	1ajq	4.31	3.54	3.68	3.62
1olu	4.41	4.27	4.39	4.26	1jaq	4.48	5.77	5.76	5.78
1hi4	4.49	5.11	5.36	4.9	1bma	4.59	7.19	7.43	7.12
1l2s	4.59	5.75	5.5	5.4	1u33	4.6	6.01	6.35	5.85
1f4f	4.62	5.74	5.75	5.94	1v2o	4.73	4.66	4.63	4.42
1sv3	4.74	3.93	3.79	4.32	1q8t	4.76	6.0	6.02	5.93
2qwd	4.85	5.04	4.67	4.65	2brb	4.86	6.11	6.0	6.03
1p1q	4.89	5.58	5.67	5.79	2j77	4.89	6.45	6.31	6.38
1e1v	4.92	5.88	5.97	5.55	2gss	4.94	4.77	4.65	5.38
1tsy	4.96	5.6	5.62	5.67	2d0k	5.02	5.88	5.89	6.24
1jqd	5.16	5.63	5.72	5.8	1rnt	5.19	5.21	5.1	4.9
1j17	5.22	7.22	7.23	7.08	1ndw	5.23	6.92	6.78	6.41
1fh7	5.24	5.83	5.74	5.98	1a69	5.3	7.4	7.24	6.3
1hk4	5.31	5.23	4.91	5.93	1gpk	5.37	6.59	6.68	6.7
1pz5	5.4	6.53	6.81	6.64	1trd	5.4	5.37	5.29	4.84
3pch	5.4	3.78	3.7	3.65	1g7f	5.47	5.8	5.83	6.14
1ha2	5.54	5.85	6.07	5.87	1vfn	5.6	4.82	4.91	5.4
1a08	5.62	6.29	6.11	6.26	1nhu	5.66	7.16	7.02	6.89
2h3e	5.7	6.77	6.8	6.3	2b1v	5.74	7.12	7.12	6.95
2flb	5.74	6.73	6.65	6.78	1ppm	5.8	7.92	7.79	7.78
1ols	5.82	5.39	5.3	4.89	3gss	5.82	6.18	6.18	6.05
1k4g	5.85	5.75	5.76	5.77	2brm	5.89	6.02	5.95	5.94
1o0h	5.92	5.73	5.57	5.22	1kv1	5.94	6.74	6.84	6.52
1b9j	5.96	6.82	6.79	7.0	1uwt	5.97	5.97	5.93	5.7
1xgj	6.0	5.41	5.16	5.41	5er1	6.02	7.21	7.22	7.23
1g7q	6.06	6.82	6.71	6.56	1m2q	6.1	6.16	6.16	6.09
1nc1	6.12	8.53	8.39	7.53	1zs0	6.15	7.52	7.57	7.0
1ndy	6.17	7.61	7.67	7.15	2fai	6.24	7.15	7.09	6.97
1pbq	6.27	5.79	5.83	5.95	1nja	6.31	5.55	5.69	5.63
1syh	6.31	5.91	5.95	5.83	1ela	6.36	6.7	6.72	6.65
1lol	6.39	4.91	4.91	6.66	10gs	6.4	6.4	6.27	6.57
1a1b	6.4	6.0	6.03	6.18	2j78	6.42	5.55	5.66	5.8
1fh9	6.43	6.2	6.13	6.08	2bzz	6.43	5.92	5.73	5.41
1f4g	6.48	6.5	6.48	6.48	2usn	6.51	7.07	6.96	7.0
1k9s	6.52	7.38	7.3	6.58	2bok	6.55	7.12	7.31	7.45
2d3z	6.64	6.42	6.45	6.57	1o3p	6.66	6.54	6.43	6.71
1om1	6.77	7.33	7.35	7.04	1fh8	6.89	5.99	6.09	6.66

PDBID	Exp BA	Pred BA[a]	Pred BA[b]	Pred BA[c]	PDBID	Exp BA	Pred BA[a]	Pred BA[b]	Pred BA[c]
1b39	6.92	5.91	5.94	6.28	2d3u	6.92	6.79	6.93	6.76
1fki	7.0	6.74	6.49	6.95	1is0	7.0	6.06	6.04	6.1
1tyr	7.0	5.03	4.84	5.94	2aov	7.04	6.14	6.07	6.15
2bz6	7.09	7.07	7.04	7.19	1elb	7.15	7.06	6.97	6.87
1fcx	7.19	8.57	8.56	8.38	1q7a	7.19	5.51	5.43	5.95
3pcj	7.22	5.91	5.88	5.42	1dhi	7.26	6.41	6.38	6.47
2ceq	7.28	6.48	6.52	6.32	2cgr	7.28	6.92	6.87	7.27
1tmn	7.3	7.3	7.28	7.27	1ydt	7.32	7.72	7.79	7.18
1ttm	7.35	7.59	7.51	8.04	1zoe	7.4	6.8	6.66	6.74
2er9	7.4	6.97	7.03	7.11	2bak	7.43	7.79	7.96	7.63
1vzq	7.44	7.62	7.58	7.59	2qwe	7.48	5.99	5.78	5.36
1d09	7.57	6.97	6.82	7.13	2fdp	7.59	8.17	8.12	8.44
1ftm	7.61	5.56	5.66	5.8	1e5a	7.64	7.24	6.99	7.05
1nny	7.66	6.96	6.83	6.63	1s39	7.7	5.1	5.18	5.19
2d1o	7.7	7.32	7.48	7.39	2aou	7.73	7.07	7.0	6.79
1u1b	7.8	6.65	6.35	6.44	1v48	7.8	6.0	5.99	6.35
1xd1	7.92	6.51	6.52	6.62	1o3f	7.96	6.56	6.52	6.79
1apw	8.0	8.32	8.29	8.38	1b7h	8.02	6.24	6.43	6.62
2cet	8.02	8.1	7.86	7.83	1gni	8.07	5.72	5.9	6.06
1fzj	8.1	7.21	7.14	7.35	1ndz	8.11	7.36	7.34	7.54
2f80	8.18	8.89	8.95	8.99	1nvq	8.25	8.29	8.26	8.06
1h23	8.35	8.73	8.58	8.73	1fd0	8.4	8.39	8.42	8.35
1fzk	8.4	7.83	7.8	7.73	2baj	8.4	8.4	8.37	7.53
1jq9	8.45	6.16	6.4	6.47	2g5u	8.49	6.73	6.69	6.76
6std	8.64	9.3	9.27	9.41	1hfs	8.7	8.25	8.15	7.92
1pxo	8.7	6.0	6.03	6.35	2b7d	8.7	7.46	7.25	7.17
1fkn	8.8	7.87	7.72	8.2	1nfy	8.89	8.06	8.09	8.05
8cpa	9.15	10.19	9.93	10.14	1sqa	9.21	7.43	7.38	7.34
1fcz	9.22	8.55	8.55	8.24	1zvx	9.22	7.38	7.46	6.91
2cer	9.22	8.15	7.9	7.57	2ayr	9.29	8.3	8.41	8.4
4er2	9.3	7.71	7.76	7.74	1re8	9.52	8.17	8.12	7.83
2g94	9.52	7.86	8.05	8.21	1df8	9.7	11.23	11.07	10.2
1fkb	9.7	8.53	8.56	8.88	2std	9.85	10.07	9.92	9.61
1e66	9.89	7.91	7.91	7.61	2drc	9.89	6.22	6.22	6.36
1bxo	10.0	7.66	7.6	7.8	1flr	10.0	6.88	6.8	7.21
4tmn	10.17	8.2	8.18	7.88	1if7	10.52	8.67	8.65	8.98
1b8o	10.64	8.48	8.52	9.35	1x1z	11.06	10.52	10.56	10.07
3std	11.11	9.47	9.34	8.97	1mq6	11.15	8.77	8.78	8.04
1y6q	11.7	7.66	8.13	7.65	1sl3	11.85	7.59	7.88	7.54
2i0d	12.1	10.6	10.3	10.6	2f01	13.0	12.81	12.83	12.9
7cpa	13.96	9.9	9.91	9.9					

E2. Results for PDBbind v2013 Dataset

Table S5 presents the PDBID, experimental binding affinities (Exp BA), and predicted binding affinities (Pred BA) obtained from the PDFL models on the PDBBind v2013 core set. The predictions include results from the one-scale (PDFL$^{L}_{3,2}$), two-scale (PDFL$^{LL}_{3,2;3,1}$), and four-scale models (PDFL$^{LLEE}_{3,2;3,1;10,3.5;6,2.5}$), corresponding to the superscripts a, b, and c in Pred BA, respectively.

Table S5: PDBbind v2013: Experimental and Predicted Binding Affinities.

PDBID	Exp BA	Pred BA[a]	Pred BA[b]	Pred BA[c]	PDBID	Exp BA	Pred BA[a]	Pred BA[b]	Pred BA[c]
3ao4	2.07	4.78	4.8	6.6	3i3b	2.23	3.6	3.47	4.75
1uto	2.27	3.27	3.19	4.58	1ps3	2.28	5.53	5.62	6.51
1qi0	2.35	3.8	3.84	3.82	3g2z	2.36	3.14	3.27	4.0
3dxg	2.4	3.89	3.86	5.76	3l7b	2.4	3.8	3.5	5.0
3mfv	2.52	5.59	5.4	6.09	3b3s	2.55	4.56	4.55	4.74
3kgp	2.57	4.44	4.31	5.73	3fk1	2.62	4.42	4.37	5.7
3fcq	2.77	4.27	4.5	4.37	3lka	2.82	5.1	4.82	6.92
3udh	2.85	4.45	4.55	5.82	4gqq	2.89	4.8	4.66	5.61
3imc	2.96	4.76	4.63	4.96	2xdl	3.1	6.38	6.42	6.87
2ymd	3.16	6.32	6.39	5.65	1lbk	3.18	4.93	4.87	5.94
1bcu	3.28	5.06	5.19	4.24	3zsx	3.28	4.06	4.16	5.85
1f8d	3.4	4.71	4.78	5.26	3muz	3.46	3.32	3.4	4.61
2v00	3.66	5.07	5.14	7.07	1loq	3.7	5.5	5.53	6.3
3n7a	3.7	4.15	4.29	5.11	2r23	3.72	5.34	4.88	5.37
3nq3	3.78	5.27	5.44	6.4	2hb1	3.8	5.33	5.16	5.25
2w66	4.05	5.71	5.49	5.91	1n2v	4.08	5.63	5.66	5.9
3kwa	4.08	6.18	5.91	6.86	3g2n	4.09	4.92	4.91	4.69
4de2	4.12	5.53	5.56	5.56	3ozt	4.13	6.28	6.27	5.96
3b3w	4.19	5.09	5.84	5.62	3cft	4.19	5.15	5.18	5.77
3f3a	4.19	6.62	6.79	6.61	2qmj	4.21	5.7	5.96	6.38
3f80	4.22	5.6	5.83	6.81	1a30	4.3	5.8	5.55	6.33
1w3k	4.3	5.02	5.15	4.43	3ivg	4.3	6.38	6.34	6.5
2jdy	4.37	5.45	5.63	6.25	3u9q	4.38	5.31	5.39	6.51
3pxf	4.43	5.12	5.28	6.36	2wbg	4.45	6.15	6.17	5.17
1u33	4.6	5.6	5.8	7.47	2x0y	4.6	5.84	5.89	8.02
3mss	4.66	4.81	5.01	6.19	1vso	4.72	5.59	5.71	7.19
1q8t	4.76	6.48	6.37	6.17	3acw	4.76	6.76	6.94	6.87
3bpc	4.8	4.95	4.91	6.43	3vd4	4.82	3.61	3.63	4.21
3cj2	4.85	5.75	5.85	6.87	2brb	4.86	6.26	6.19	6.1
1p1q	4.89	7.07	6.99	5.94	2vo5	4.89	6.73	7.1	5.88
3d4z	4.89	5.54	5.17	4.9	2gss	4.94	4.86	4.92	5.66
2yge	5.06	6.43	6.29	5.99	3gy4	5.1	4.43	4.4	5.26
3zso	5.12	5.75	5.86	5.99	3ov1	5.2	5.62	5.65	6.09
1w4o	5.22	4.66	4.71	4.06	1zea	5.22	6.5	6.38	7.23
2zxd	5.22	5.59	5.51	6.39	3ueu	5.24	5.54	5.6	6.1
2qft	5.26	4.5	4.37	5.76	1gpk	5.37	6.95	6.96	8.28
1f8b	5.4	3.9	3.97	3.91	2jdm	5.4	5.9	5.79	7.3
3su5	5.58	6.56	6.54	6.61	2wca	5.6	5.78	5.24	5.78
3n86	5.64	7.14	7.0	6.42	2x97	5.66	7.48	7.33	7.06
1n1m	5.7	4.91	5.18	6.17	1o5b	5.77	6.11	5.99	6.91
2y5h	5.79	7.62	7.67	7.33	3ehy	5.85	6.24	6.45	7.45
4des	5.85	6.49	6.35	6.09	3ebp	5.91	5.46	5.58	4.83
1q8u	5.96	6.53	6.48	6.95	4de1	5.96	5.51	5.54	5.52
3huc	5.99	6.38	6.64	7.0	3l4w	6.0	5.57	5.46	5.54
2vl4	6.01	5.36	5.45	5.86	3coy	6.02	6.03	6.07	6.1
3f3c	6.02	6.24	6.43	6.47	1os0	6.03	8.17	8.18	6.76
3owj	6.07	6.67	6.83	7.1	3bkk	6.08	6.83	6.59	5.89
1yc1	6.17	7.68	7.62	6.5	1hnn	6.24	6.65	6.73	6.83
3vh9	6.24	4.5	4.8	5.42	3bfu	6.27	6.43	6.43	7.07
1w3l	6.28	5.42	5.81	5.55	3k5v	6.3	6.87	6.75	5.6

PDBID	Exp BA	Pred BA[a]	Pred BA[b]	Pred BA[c]	PDBID	Exp BA	Pred BA[a]	Pred BA[b]	Pred BA[c]
2qbr	6.33	7.07	7.01	7.34	1lol	6.39	5.12	4.88	6.04
10gs	6.4	6.07	5.82	6.19	2j78	6.42	5.84	5.93	5.99
1r5y	6.46	5.72	6.0	6.38	2weg	6.5	6.76	6.65	7.5
3uo4	6.52	7.35	7.61	7.49	3jvs	6.54	6.05	5.99	5.89
2yfe	6.63	7.28	7.43	6.86	1sln	6.64	7.31	7.42	7.54
2iwx	6.68	7.13	7.12	6.79	2jdu	6.72	5.96	5.94	5.69
4djv	6.72	7.47	7.47	6.75	2xhm	6.8	7.92	7.87	6.69
2xnb	6.83	8.44	8.16	7.81	3s8o	6.85	6.23	6.27	5.61
2zcr	6.87	7.61	7.66	6.38	3oe5	6.88	6.43	6.38	5.97
3gbb	6.9	7.16	7.28	7.16	2d3u	6.92	6.61	6.68	6.61
3uex	6.92	6.74	6.7	6.78	4dew	7.0	5.92	5.86	7.16
1xd0	7.12	5.23	5.33	6.48	1z95	7.12	8.51	8.41	6.36
2vot	7.14	8.0	7.68	7.55	1oyt	7.24	7.14	7.15	6.82
2ole	7.25	7.0	6.86	5.69	3gcs	7.25	7.8	7.87	7.19
1kel	7.28	6.53	6.22	6.49	2vvn	7.3	6.57	6.61	7.25
3kv2	7.32	5.63	5.59	6.57	3pww	7.32	7.05	7.26	7.46
3su2	7.35	6.73	6.7	6.04	1f8c	7.4	4.92	5.07	5.19
2xys	7.42	7.03	7.01	7.25	3l4u	7.52	5.15	5.43	6.13
2xb8	7.59	7.14	7.33	5.03	2d1o	7.7	7.62	7.63	6.77
2zjw	7.7	5.82	5.99	6.38	3f3e	7.7	6.19	6.4	5.72
2g70	7.77	6.55	6.44	6.83	2zwz	7.79	6.07	6.31	6.17
1u1b	7.8	6.4	6.01	5.49	4g8m	7.89	6.09	6.37	5.91
1o3f	7.96	6.59	6.51	6.62	2x8z	7.96	6.61	6.5	7.45
3cyx	8.0	8.38	8.36	6.07	2cet	8.02	7.72	7.79	5.59
3ag9	8.05	5.67	5.83	6.9	2pq9	8.11	6.45	6.59	6.35
3l3n	8.18	6.5	6.71	5.75	1nvq	8.25	8.17	8.15	7.8
2cbj	8.27	5.95	5.8	6.76	2v7a	8.3	7.94	7.56	7.38
1h23	8.35	8.7	8.61	7.51	2qbp	8.4	6.38	6.34	5.49
3b68	8.4	7.83	8.02	6.9	2xbv	8.43	8.22	8.09	6.74
2fvd	8.52	7.81	7.83	8.06	2vw5	8.52	6.83	6.83	6.8
3ejr	8.57	7.79	7.84	8.05	3f17	8.63	7.02	7.2	7.51
3nox	8.66	7.8	7.71	7.07	1hfs	8.7	8.18	8.29	6.56
1jyq	8.7	6.18	6.37	5.93	2pcp	8.7	6.09	6.12	6.54
3ge7	8.7	7.46	7.48	6.63	2wtv	8.74	8.06	8.03	6.09
2zcq	8.82	6.4	6.62	5.65	2obf	8.85	7.47	7.53	5.66
3e93	8.85	8.29	8.3	6.44	2p4y	9.0	8.55	8.59	7.98
3dd0	9.0	8.84	8.82	6.93	3nw9	9.0	8.25	8.26	7.0
3uri	9.0	7.11	7.05	7.22	3gnw	9.1	7.73	7.78	7.29
3su3	9.13	6.6	6.67	6.49	2xy9	9.19	7.18	7.22	6.67
1sqa	9.21	7.22	7.13	6.79	3fv1	9.3	8.42	8.46	6.67
2yki	9.46	7.54	7.66	7.16	3g0w	9.52	8.26	8.31	7.43
3pe2	9.76	8.13	8.34	6.16	1e66	9.89	7.43	7.61	7.21
1igj	10.0	6.56	6.51	5.46	4tmn	10.17	7.27	7.35	6.73
2zx6	10.6	8.12	8.22	7.89	3myg	10.7	6.54	6.55	6.8
4gid	10.77	8.28	8.45	6.7	3utu	10.92	8.25	8.27	8.0
1lor	11.06	8.61	8.58	7.84	1mq6	11.15	8.81	8.76	7.64
2x00	11.33	7.41	7.4	7.11	2j62	11.34	8.02	8.21	6.9
4djr	11.52	9.84	9.73	8.43					

E3. Results for PDBbind v2016 Dataset

Table S6 provides the PDBID, experimental binding affinities (Exp BA), and predicted binding affinities (Pred BA) for the PDBBind v2016 core set. Predictions are reported for the one-scale ($\mathrm{PDFL}^{L}_{3,2}$), two-scale ($\mathrm{PDFL}^{LL}_{3,2;3,1}$), and four-scale models ($\mathrm{PDFL}^{LLEE}_{3,2;3,1;10,3.5;6,2.5}$), with superscripts a, b, and c in Pred BA indicating the corresponding model scales.

Table S6: PDBbind v2016: Experimental and Predicted Binding Affinities.

PDBID	Exp BA	Pred BA^a	Pred BA^b	Pred BA^c	PDBID	Exp BA	Pred BA^a	Pred BA^b	Pred BA^c
3ao4	2.07	4.48	4.96	4.059	3gv9	2.12	4.76	4.61	4.207
1uto	2.27	3.55	3.39	3.501	1ps3	2.28	5.51	5.86	5.575
4ddk	2.29	3.68	3.51	3.113	4jsz	2.3	5.28	5.2	5.367
3g2z	2.36	2.89	3.51	3.592	3dxg	2.4	3.61	4.17	3.859
3l7b	2.4	4.0	3.72	3.57	3gr2	2.52	3.07	3.74	3.769
3kgp	2.57	4.0	4.08	3.872	3fcq	2.77	3.89	4.36	4.695
3lka	2.82	4.98	4.4	5.716	3zt2	2.84	3.34	3.61	3.538
3udh	2.85	4.35	4.47	4.519	3g31	2.89	4.18	3.98	4.294
4llx	2.89	4.52	4.02	4.059	4u4s	2.92	5.22	5.4	5.36
4owm	2.96	4.1	3.89	4.082	5aba	2.98	4.74	5.11	4.889
2xdl	3.1	6.2	6.31	6.376	4kz6	3.1	4.46	4.04	3.689
2ymd	3.16	6.19	6.69	6.225	3aru	3.22	5.32	5.22	5.303
1bcu	3.28	4.96	5.08	5.049	3zsx	3.28	3.69	4.09	3.909
4ddh	3.3	4.81	4.86	4.299	4mrw	3.3	4.38	4.06	4.156
4eky	3.52	4.89	5.1	4.882	4mrz	3.52	4.33	4.02	3.986
4abg	3.57	4.36	4.33	4.312	5a7b	3.57	5.13	5.42	5.292
3dx1	3.58	6.46	6.22	5.916	4bkt	3.62	4.6	4.86	4.588
2v00	3.66	4.85	5.29	5.01	4cig	3.67	4.35	4.52	3.933
3n7a	3.7	3.97	4.28	4.577	3d6q	3.76	4.04	4.1	4.033
2hb1	3.8	5.01	4.85	4.738	3twp	3.92	4.14	4.05	4.175
4agn	3.97	4.76	4.94	4.691	1c5z	4.01	4.14	4.27	4.265
3nq9	4.03	3.97	4.5	4.16	4msn	4.03	3.83	3.81	3.565
2w66	4.05	5.46	5.43	5.352	3kwa	4.08	6.02	6.01	5.909
3g2n	4.09	5.07	4.73	4.646	4cr9	4.1	4.67	4.88	4.934
4ih5	4.11	4.95	5.28	4.984	4de2	4.12	5.45	5.44	5.226
3ozt	4.13	6.34	6.6	6.217	3f3a	4.19	6.12	6.69	6.169
1a30	4.3	6.6	5.76	6.527	3ivg	4.3	6.84	6.87	6.044
3u9q	4.38	5.2	5.28	5.18	3rsx	4.41	4.86	5.12	4.878
3pxf	4.43	4.78	4.69	5.282	2wbg	4.45	6.05	6.44	6.06
3rr4	4.55	6.4	6.57	6.297	4w9c	4.65	4.92	5.2	4.994
3mss	4.66	4.7	4.84	5.565	4agp	4.69	5.04	5.27	5.064
4mgd	4.69	7.69	7.16	6.97	1vso	4.72	5.22	5.19	5.978
4jxs	4.74	5.11	5.21	4.578	1q8t	4.76	6.69	6.4	6.01
3acw	4.76	6.54	6.95	6.718	4lzs	4.8	4.67	5.02	5.08
3r88	4.82	4.25	4.8	4.59	4ciw	4.82	4.49	4.73	5.09
2w4x	4.85	5.33	5.35	5.037	2brb	4.86	6.81	6.88	6.551
1p1q	4.89	7.07	6.58	6.853	3d4z	4.89	5.48	5.55	5.916
1bzc	4.92	5.88	5.6	5.66	1nc3	5.0	5.32	5.68	5.892
4agq	5.01	5.09	5.37	5.065	4w9l	5.02	6.34	6.42	6.399
2yge	5.06	6.42	6.49	6.182	5c1w	5.06	5.14	5.06	5.565
2r9w	5.1	5.28	5.66	5.16	3gy4	5.1	4.38	4.6	4.641
3syr	5.1	4.14	4.44	4.318	3zso	5.12	5.01	5.67	5.04

PDBID	Exp BA	Pred BA[a]	Pred BA[b]	Pred BA[c]	PDBID	Exp BA	Pred BA[a]	Pred BA[b]	Pred BA[c]
2br1	5.14	7.25	7.14	7.262	1s38	5.15	6.11	6.25	5.891
3b27	5.16	6.0	6.14	6.119	4gkm	5.17	5.49	5.34	5.046
4m0z	5.19	6.51	6.6	6.447	1w4o	5.22	4.66	4.71	4.508
3ueu	5.24	4.97	4.94	4.66	4ih7	5.24	5.68	5.76	5.319
4jfs	5.27	5.87	5.38	6.01	3ozs	5.33	6.2	6.72	6.288
3bv9	5.36	7.54	7.08	7.298	1gpk	5.37	6.84	7.01	7.047
1syi	5.44	6.85	6.65	6.554	2cbv	5.48	5.35	5.84	5.77
1ydr	5.52	6.42	6.46	6.475	4de3	5.52	5.21	5.26	5.039
3coz	5.57	5.9	5.83	5.722	2wca	5.6	6.1	5.45	5.937
3u5j	5.61	5.87	5.79	6.052	4dli	5.62	6.18	6.34	6.322
1z9g	5.64	5.33	5.09	5.335	3arv	5.64	5.7	5.82	5.925
3n86	5.64	6.67	6.43	6.44	5c28	5.66	4.84	4.57	5.035
4j28	5.7	6.21	6.03	6.766	3jvr	5.72	4.66	5.36	6.099
1o5b	5.77	6.11	5.32	6.005	2y5h	5.79	7.62	7.36	7.411
3qqs	5.82	5.49	5.41	5.253	3wz8	5.82	6.63	6.91	6.378
4dld	5.82	5.89	6.02	6.069	3ehy	5.85	5.7	6.05	6.408
3uev	5.89	5.94	5.79	5.137	3ebp	5.91	5.57	5.79	5.753
1o0h	5.92	5.49	5.55	5.281	1q8u	5.96	6.72	6.75	6.429
4de1	5.96	5.52	5.53	5.242	4msc	5.96	6.35	6.15	6.128
4w9i	5.96	5.83	5.79	5.659	3ary	6.0	4.61	4.45	5.126
3coy	6.02	6.19	6.18	5.997	3f3c	6.02	5.26	5.91	5.601
2fxs	6.06	6.48	6.61	6.73	4kzq	6.1	6.56	5.96	6.078
2qnq	6.11	7.58	7.44	7.049	1nc1	6.12	7.79	7.89	7.542
2wvt	6.12	5.09	5.24	6.056	1yc1	6.17	7.59	7.16	7.497
3bgz	6.26	7.03	7.06	6.96	4wiv	6.26	6.42	6.43	6.466
3k5v	6.3	6.66	7.22	7.22	4eor	6.3	7.64	7.74	7.905
3uew	6.31	6.37	5.56	5.388	2wnc	6.32	6.48	6.31	6.989
2zb1	6.32	7.8	7.78	7.707	2qbr	6.33	6.67	6.35	6.514
3arq	6.4	6.22	6.06	6.313	2j78	6.42	5.44	5.93	5.722
4ea2	6.44	6.77	6.96	6.838	1r5y	6.46	5.56	5.46	5.457
4m0y	6.46	6.09	6.49	6.427	1gpn	6.48	6.97	6.92	7.16
2weg	6.5	7.07	6.65	6.592	4kzu	6.5	6.63	6.03	6.08
4mme	6.5	7.19	7.04	6.907	3cj4	6.51	5.73	5.65	5.789
3uo4	6.52	7.48	7.19	7.252	3wtj	6.53	7.61	7.63	7.639
3jvs	6.54	5.9	6.15	6.452	1k1i	6.58	6.61	6.83	6.615
2yfe	6.63	6.46	7.04	6.683	4k77	6.63	7.26	7.27	7.357
2xj7	6.66	5.38	5.36	5.255	2iwx	6.68	6.98	6.91	6.853
4f09	6.7	7.05	6.92	7.205	4djv	6.72	7.3	7.04	6.936
4w9h	6.73	6.15	6.23	6.063	4ogj	6.79	6.77	6.59	6.906
1p1n	6.8	6.64	6.3	6.345	3dx2	6.82	6.84	6.02	6.129
2xnb	6.83	8.46	7.79	7.833	3n76	6.85	6.37	6.31	6.373
3pyy	6.86	6.17	6.43	6.866	2zcr	6.87	7.56	7.56	7.38
3oe5	6.88	6.3	6.68	6.284	3jya	6.89	5.82	6.25	6.42
3gbb	6.9	7.13	6.3	6.847	3uex	6.92	6.51	6.32	5.867
4f9w	6.94	6.99	6.93	6.866	2wer	7.05	7.68	6.98	7.339
1lpg	7.09	7.96	7.93	7.819	3zdg	7.1	6.32	6.66	6.548
1z95	7.12	8.16	7.8	8.009	1pxn	7.15	7.67	7.32	7.209
3arp	7.15	6.8	6.73	6.805	3f3d	7.16	6.24	6.2	6.112
3tsk	7.17	7.4	7.96	8.072	2j7h	7.19	6.48	6.27	6.548
2xii	7.2	7.04	6.8	7.361	4cra	7.22	8.05	7.73	7.861
4gfm	7.22	6.63	6.94	6.916	1oyt	7.24	7.31	7.62	7.583

PDBID	Exp BA	Pred BA[a]	Pred BA[b]	Pred BA[c]	PDBID	Exp BA	Pred BA[a]	Pred BA[b]	Pred BA[c]
3p5o	7.26	7.04	6.69	6.78	3gc5	7.26	6.98	6.68	6.641
2vvn	7.3	6.36	6.4	6.232	1qf1	7.32	7.17	7.44	6.904
1ydt	7.32	7.62	7.69	7.185	3pww	7.32	7.04	7.9	7.201
1owh	7.4	6.6	6.59	6.823	2zy1	7.4	6.82	6.79	6.531
3up2	7.4	7.65	7.36	7.417	4j21	7.41	7.09	7.05	7.096
2xys	7.42	6.98	6.74	7.068	2qbq	7.44	6.56	6.13	6.518
3oe4	7.47	6.1	6.91	6.299	3rlr	7.52	7.57	7.47	7.637
2xb8	7.59	6.69	6.36	6.589	2c3i	7.6	6.9	7.02	6.812
4e5w	7.66	8.32	7.99	8.45	3f3e	7.7	5.89	5.82	5.799
1u1b	7.8	6.86	6.0	6.14	3qgy	7.8	6.56	7.21	6.809
3ryj	7.8	7.55	7.71	7.875	4j3l	7.8	7.78	7.65	7.602
3prs	7.82	7.95	7.88	7.595	4pcs	7.85	6.16	5.4	6.438
4hge	7.92	7.74	7.88	7.857	1o3f	7.96	6.37	6.33	6.49
2qe4	7.96	6.72	7.43	7.627	3uuo	7.96	6.75	6.4	7.055
3cyx	8.0	8.03	7.81	7.773	3e92	8.0	7.75	7.96	6.931
3fur	8.0	7.84	8.13	7.639	2cet	8.02	7.45	7.01	7.594
5tmn	8.04	7.01	7.03	7.173	3ag9	8.05	6.35	6.8	6.744
3kr8	8.1	6.83	7.1	6.777	3nx7	8.1	7.63	7.02	7.686
3fv2	8.11	7.71	6.57	7.027	4eo8	8.15	7.0	7.15	7.307
3e5a	8.23	7.62	7.3	7.644	1nvq	8.25	8.36	8.11	8.174
2v7a	8.3	8.13	7.11	7.796	4x6p	8.3	8.38	8.34	8.391
1h23	8.35	7.62	7.34	7.577	4e6q	8.36	7.66	7.66	7.971
2al5	8.4	6.74	6.39	6.563	2qbp	8.4	6.43	6.45	6.583
2zda	8.4	6.77	7.01	6.643	3b68	8.4	7.71	8.1	8.21
2xbv	8.43	8.3	8.19	8.007	3b1m	8.48	7.96	7.82	7.641
2fvd	8.52	7.98	7.82	8.04	2vw5	8.52	7.04	6.89	7.003
2wn9	8.52	6.01	5.99	6.513	3ejr	8.57	7.75	7.44	7.748
4qd6	8.64	6.01	5.48	6.584	3u8k	8.66	7.21	7.04	7.407
3ge7	8.7	7.22	7.75	7.248	4crc	8.72	8.41	8.3	8.168
4ivb	8.72	8.2	8.18	8.181	2vkm	8.74	8.67	8.33	8.322
2wtv	8.74	8.24	8.01	7.823	3b5r	8.77	8.0	7.75	8.216
2zcq	8.82	6.6	6.53	6.985	3e93	8.85	8.23	8.31	7.087
4k18	8.96	7.89	7.79	7.718	2p4y	9.0	7.82	8.15	7.821
3dd0	9.0	8.41	7.69	8.066	3nw9	9.0	8.04	7.67	8.064
3ui7	9.0	6.88	7.15	7.327	3uri	9.0	7.22	6.88	6.93
1qkt	9.04	7.04	7.03	7.705	1h22	9.1	7.63	7.64	7.652
3gnw	9.1	7.86	7.46	7.331	1sqa	9.21	7.23	7.05	7.375
4jia	9.22	7.4	7.28	7.641	3b65	9.27	7.87	7.82	8.014
3fv1	9.3	8.4	7.38	8.28	4qac	9.4	7.52	7.56	7.353
2yki	9.46	7.54	7.96	7.569	3g0w	9.52	7.75	8.13	8.255
4ivd	9.52	8.1	8.19	8.328	4ty7	9.52	7.95	7.57	7.936
2pog	9.54	6.89	7.39	7.483	4gr0	9.55	9.01	8.79	8.619
1eby	9.7	8.83	9.12	9.079	1z6e	9.72	8.63	9.06	8.92
1e66	9.89	6.75	7.2	6.779	4ivc	10.0	8.34	8.4	8.345
4twp	10.0	8.17	7.8	8.237	4rfm	10.05	8.23	7.99	8.051
1y6r	10.11	8.54	8.93	8.81	3u8n	10.17	7.32	7.63	7.451
4tmn	10.17	7.24	7.14	7.221	2p15	10.3	8.39	9.11	8.916
3myg	10.7	6.71	6.75	7.22	4gid	10.77	8.24	8.23	8.174
3utu	10.92	8.37	8.23	7.939	5c2h	11.09	7.57	7.55	7.933
1mq6	11.15	8.81	8.48	8.754	5dwr	11.22	7.96	7.99	8.041
4f2w	11.3	10.26	10.07	10.235	2x00	11.33	7.48	7.42	7.661

**PDBID**	**Exp BA**	**Pred BA**[a]	**Pred BA**[b]	**Pred BA**[c]	**PDBID**	**Exp BA**	**Pred BA**[a]	**Pred BA**[b]	**Pred BA**[c]
3o9i	11.82	10.45	10.03	10.518	4f3c	11.82	9.88	9.96	9.803

References

[1] Kelin Xia and Guo-Wei Wei. Persistent homology analysis of protein structure, flexibility, and folding. *International journal for numerical methods in biomedical engineering*, 30(8):814–844, 2014.

[2] Zixuan Cang, Lin Mu, Kedi Wu, Kristopher Opron, Kelin Xia, and Guo-Wei Wei. A topological approach for protein classification. *Computational and Mathematical Biophysics*, 3(1), 2015.

[3] Robert Ghrist. Barcodes: the persistent topology of data. *Bulletin of the American Mathematical Society*, 45(1):61–75, 2008.

[4] Zixuan Cang and Guo-Wei Wei. Integration of element specific persistent homology and machine learning for protein-ligand binding affinity prediction. *International journal for numerical methods in biomedical engineering*, 34(2):e2914, 2018.

[5] Duc D Nguyen, Tian Xiao, Menglun Wang, and Guo-Wei Wei. Rigidity strengthening: A mechanism for protein–ligand binding. *Journal of chemical information and modeling*, 57(7):1715–1721, 2017.

[6] Kristopher Opron, Kelin Xia, and Guo-Wei Wei. Fast and anisotropic flexibility-rigidity index for protein flexibility and fluctuation analysis. *The Journal of chemical physics*, 140(23), 2014.

[7] Kelin Xia, Kristopher Opron, and Guo-Wei Wei. Multiscale multiphysics and multidomain models—flexibility and rigidity. *The Journal of chemical physics*, 139(19), 2013.

[8] Benjamin Jones and Guo-Wei Wei. Persistent directed flag laplacian. *Foundations of Data Science*, accepted 2024.

[9] Daniel Lütgehetmann, Dejan Govc, Jason P Smith, and Ran Levi. Computing persistent homology of directed flag complexes. *Algorithms*, 13(1):19, 2020.

[10] Jie Liu and Renxiao Wang. Classification of current scoring functions. *Journal of chemical information and modeling*, 55(3):475–482, 2015.

[11] Fabian Pedregosa, Gaël Varoquaux, Alexandre Gramfort, Vincent Michel, Bertrand Thirion, Olivier Grisel, Mathieu Blondel, Peter Prettenhofer, Ron Weiss, Vincent Dubourg, et al. Scikit-learn: Machine learning in python. *the Journal of machine Learning research*, 12:2825–2830, 2011.
